# Endothelial PDGF-D contributes to neurovascular protection after ischemic stroke by rescuing pericyte functions

**DOI:** 10.1007/s00018-024-05244-w

**Published:** 2024-05-21

**Authors:** Maxime Bernard, Romain Menet, Sarah Lecordier, Ayman ElAli

**Affiliations:** 1 Department of Psychiatry and Neuroscience, Faculty of Medicine, Université Laval, Quebec City, QC Canada; 2 Neuroscience Axis, Research Center of CHU de Québec (CHUQ)-Université Laval, 2705 Laurier Boulevard, Quebec City, QC G1V 4G2 Canada

**Keywords:** Ischemic stroke, Pericytes, PDGF-D, Neurovascular functions, Angiogenesis, Repair

## Abstract

Ischemic stroke induces neovascularization of the injured tissue as an attempt to promote structural repair and neurological recovery. Angiogenesis is regulated by pericytes that potently react to ischemic stroke stressors, ranging from death to dysfunction. Platelet-derived growth factor (PDGF) receptor (PDGFR)β controls pericyte survival, migration, and interaction with brain endothelial cells. PDGF-D a specific ligand of PDGFRβ is expressed in the brain, yet its regulation and role in ischemic stroke pathobiology remains unexplored. Using experimental ischemic stroke mouse model, we found that PDGF-D is transiently induced in brain endothelial cells at the injury site in the subacute phase. To investigate the biological significance of PDGF-D post-ischemic stroke regulation, its subacute expression was either downregulated using siRNA or upregulated using an active recombinant form. Attenuation of PDGF-D subacute induction exacerbates neuronal loss, impairs microvascular density, alters vascular permeability, and increases microvascular stalling. Increasing PDGF-D subacute bioavailability rescues neuronal survival and improves neurological recovery. PDGF-D subacute enhanced bioavailability promotes stable neovascularization of the injured tissue and improves brain perfusion. Notably, PDGF-D enhanced bioavailability improves pericyte association with brain endothelial cells. Cell-based assays using human brain pericyte and brain endothelial cells exposed to ischemia-like conditions were applied to investigate the underlying mechanisms. PDGF-D stimulation attenuates pericyte loss and fibrotic transition, while increasing the secretion of pro-angiogenic and vascular protective factors. Moreover, PDGF-D stimulates pericyte migration required for optimal endothelial coverage and promotes angiogenesis. Our study unravels new insights into PDGF-D contribution to neurovascular protection after ischemic stroke by rescuing the functions of pericytes.

## Introduction

Ischemic stroke is a main leading cause of death and disability of adults worldwide. Still no disease-modifying therapy exists, and recanalization through thrombolysis or thrombectomy represents the only strategy used in clinics to restore cerebral blood flow (CBF) [1]. This therapeutic void has directed research to explore new strategies that take into consideration the complex responses at the blood-brain interface. Indeed, brain functioning depends upon the concerted crosstalk among neuronal, glial, and vascular components to generate neurovascular functions that include regulation of blood-brain barrier (BBB) properties, CBF, angiogenesis, vascular homeostasis, as well as immunomodulation [2, 3]. Deregulation of neurovascular functions after ischemic stroke decisively affects injury progression and repair, and thus influences definite lesion maturation [4–6]. Initial acute injury associated with irreversible tissue loss and subacute secondary injury associated with reperfusion, jointly determine definite lesion extension and neurological outcomes [7]. In the subacute phase, endogenous vascular protective processes are activated as an attempt to promote the survival of injured tissue [8, 9]. Indeed, various pro-angiogenic factors, namely vascular endothelial growth factor (VEGF), are induced after ischemic stroke [10]. VEGF elevated levels associated with an enhanced formation of collaterals, correlate with mild neurological deficits upon ischemic stroke occurrence [11, 12]. These observations suggest that interventions aiming to improve tissue vascularization constitute a promising therapeutic approach to promote structural repair and neurological recovery. Indeed, exogenous VEGF ameliorates outcomes after stroke by promoting angiogenesis [10, 13]. However, VEGF-based therapies constitute a double-edged sword strategy, as it increases the risk of serious complications, namely hemorrhages caused by vascular destabilization, thus hampering its clinical translation [14]. If therapeutic angiogenesis was to be translated into clinic, it is essential to develop novel approaches that enable promotion neovascularization while preserving vascular structural and functional integrity.

Brain pericytes narrowly interact with endothelial cells to regulate BBB formation, angiogenesis, and vascular functions [3]. Ischemic stroke induces rapid pericyte loss at the intralesional infarct core and detachment from endothelial cells in the perilesional region, leading to vascular destabilization that contributes to secondary injury progression [15–18]. Targeting pericytes represents a promising avenue that has the potential of improving functional vascularization of the injured tissue after ischemic stroke [12, 19–21]. Pericytes ubiquitously express platelet-derived growth factor receptor (PDGFR)β that controls cell survival, proliferation, and interaction with endothelial cells [3, 22]. PDGFRβ expression is induced after ischemic stroke, and contributes to subacute tissue structural repair [19, 22–24]. PDGF-B released by angiogenic endothelial cells activates PDGFRβ to promote pericyte recruitment to newly formed vasculature and their subsequent stabilization [22]. This suggests that pericyte functions could be targeted through modulation of PDGFRβ signaling. However, PDGF-B could activate PDGFRα, which has been shown to exacerbate vascular permeability after ischemic stroke by activating perivascular astrocytes [25, 26]. PDGF-D was recently identified to specifically activate PDGFRβ homodimers [27, 28], outlining its potential in modulating the functions of pericytes through PDGFRβ. In the healthy brain, PDGF-D seems to be mainly expressed in endothelial cells [29, 30], yet its regulation and role in ischemic stroke remains unexplored.

Herein, our study aims to elucidate the role of PDGF-D in ischemic stroke pathobiology and assess its therapeutic potential as modulator of angiogenesis. For this purpose, genetic and pharmacological approaches were employed to manipulate PDGF-D expression in an experimental ischemic stroke model, in combination with cell-based assays. Our findings indicate that PDGF-D is transiently induced in brain endothelial cells after ischemic stroke. Our data indicate that PDGF-D contributes to the maintenance of neurovascular functions by promoting the vascular protective properties of pericytes. Our results unravel a previously undescribed role of PDGF-D in stroke pathobiology and outline its therapeutic potential to promote neovascularization and neurovascular repair by acting directly on pericytes.

## Materials and methods

### Animal experiments

Three to 5 months old C57BL6/J male mice obtained from The Jackson Laboratory (# 000664, Bar Harbor, ME, USA) were used. Age-matching transgenic mice heterozygous for PDGFRβ^tm1.1(cre/ERT2)Csln^/J (PDGFRβ^CreERT2^; #030201, The Jackson Laboratory) and homozygous for B6.CG-Gt(Rosa)26Sor^tm9(CAG−tdTomato)Hze^ (Ai9; #007909, The Jackson Laboratory) were used to track PDGFRβ^+^ cells in the brain. In PDGFRβ^CreERT2^:Ai9 mice a *loxP*-flanked STOP cassette, which prevents the transcription of a CAG promoter-driven tdTomato, is inserted into Gt(ROSA)26Sor locus. All transgenic mice were maintained on a C57BL6/J background. Genotypes were confirmed by PCR using DNA isolated from ear punches. The following probes were used; For PDGFRβ^CreERT2^: Common: 5’ – CAC AAC TGA AGT AAG TTC CAC C – 3’; Wild type reverse: 5’ – GTC GAT GTG ACG GTGT TTC GA – 3’; Mutant reverse: 5’ – CGT TCT TGG ACT ACC TGT ACA – 3’. For Ai9: oIMR9020: Wild type forward: 5’ – AAG GGA GCT GCA GTG GAG TA – 3’; oIMR9021: Wild type reverse: 5’ – CCG AAA ATC TGT GGG AAG TC – 3’; oIMR9103: Mutant reverse: 5’ – GGC ATT AAA GCA GCG TAT CC – 3’; oIMR9105: Mutant forward: 5’ – CTG TTC CTG TAC GGC ATG G – 3’. Mice were housed and acclimated to standard laboratory conditions (12 h light/dark cycle; Light phase from 7:00 AM to 7:00 PM) with free access to chow and water [31]. Mice were subjected to ischemic stroke via transient intraluminal middle cerebral artery occlusion (MCAo), as described [32]. Briefly, mice were anesthetized with isoflurane (3% for induction and 1.5% for maintenance) in 95% O_2_, 2 L/min, and body temperature was maintained between 36 and 37 °C using a feedback-controlled heating system (Harvard Apparatus^®^, QC, Canada). After midline neck incision, left common and external carotid arteries were isolated and ligated. A microvascular clip was placed on the internal carotid artery (ICA), and a 7 − 0 silicon-coated nylon monofilament (Doccol Corporation, MA, USA) was directed through the ICA until MCA origin. The monofilament was left in place for 30 min and reperfusion was achieved by gently withdrawing it. Laser Doppler flowmeter (LDF) (OMEGAFLOW FLO-N1; Omegawave Inc., Japan) was used to monitor brain perfusion using a flexible fiber optic probe attached to the skull overlaying the MCA territory to confirm occlusion [32]. Animal procedures and handling were performed according to the Canadian Council on Animal Care guidelines, as implemented by *Comité de Protection des Animaux de l’Université Laval-3* (CPAUL-3; Protocol # 20–470). Animal studies were reported according to ARRIVE 2.0 guidelines.

### Cre-recombinase activation by tamoxifen

PDGFRβ^+^ cells were traced by crossbreeding PDGFRβ^CreERT2^ mice with Ai9 mice to generate PDGFRβ^CreERT2^:Ai9 transgenic mice. Cre-recombination was induced in PDGFRβ^CreERT2^:Ai9 mice to mediate excision of the *loxP*-flanked STOP cassette and the subsequent specific expression of tdTomato in PDGFRβ^+^ pericytes (PDGFRβ^tdTomato^ mice) using tamoxifen (Sigma-Aldrich, ON, Canada) dissolved at 20 mg/mL in corn oil (Sigma). Mice were injected intraperitoneally with tamoxifen (80 mg/kg; 1x day; 2 consecutive days). Three days after last injection, mice were subjected to MCAo and euthanized 1 week later.

### siRNA PDGF-D and recombinant active PDGF-D intranasal delivery

In a first set of experiments, PDGF-D expression in the brain was downregulated using siRNA. Briefly, PDGF-D Stealth® siRNA (Thermo Fisher Scientific Inc., QC, Canada) was suspended in sterile diethyl pyrocarbonate (DEPC) water. siRNA suspension was diluted in 10% glucose and sterile water. siRNA dilution was mixed with a solution of in vivo-jetPEI (N/P ratio = 8; Polyplus, NY, USA) priorly diluted in 10% glucose. The final solution was incubated for 15 min at room temperature (RT). Thirty minutes prior to siRNA infusion, mice were intranasally treated with 15 µL hyaluronidase (100U; Sigma) to improve siRNA absorption and delivery into the brain through permeabilization of the olfactory epithelium [33]. Mice (*n* = 5 per group) were intranasally infused with 15 µL of (i) vehicle, or (ii) PDGF-D siRNA (5 µg/mouse) 24 h prior to MCAo once a day and repeated every 2^nd^ day for 1 week. In a second set of experiments, PDGF-D bioavailability in the brain was increased through the delivery of active recombinant human (rh)-PDGF-D resuspended in 4 mM HCl (R&D Systems, ON, Canada). Mice (*n* = 8 per group) were intranasally infused with 15 µL of either, (i) vehicle (VEH), or (ii) rh-PDGF-D (125 or 250 ng/kg, P125 and P250, respectively), starting at 24 h after MCAo to target the subacute phase, repeated every 2^nd^ day for 1 week and a sub-group of mice were kept alive 1 week after last infusion. siRNA delivery was well tolerated by mice reflected by unchanged weight and mortality rate throughout the study.

### Brain tissue processing

For histochemical and immunohistochemical analyses, mice were euthanized via a transcardiac perfusion with 0.9% NaCl followed by 1% paraformaldehyde (PFA). Brains were retrieved and post-fixed in 4% PFA for 24 h and transferred into PFA containing 20% sucrose for 24 h at 4 °C. Brains were then cut into 25 μm coronal sections using a freezing microtome (Leica Biosystems, ON, Canada), and serial sections were collected in a 12 well-plates filled with an anti-freeze solution (30% glycerol, 30% ethylene glycol in 0.9% NaCl, phosphate buffer (PB)) and kept at -20 °C until further use. For Western blot analysis, mice were euthanized via a transcardiac perfusion with 0.9% NaCl, and brains were next quickly removed, snaped frozen on dry ice and kept at -80 °C until further use.

### Assessment of brain injury size and BBB breakdown

Free-floating PFA-fixed brain sections were mounted onto SuperFrost^®^ Plus slides (Fisher Scientific, ON, Canada) and thoroughly dried overnight under vacuum at RT. Sections were next stained with 0.5% cresyl violet to assess the infarct size and atrophy at week 1 and 2 post-occlusion, as previously described [34]. Stained sections were digitized and the borderline between injured and non-injured tissue was outlined with FIJI software (National Institutes of Health, MD, USA). Immunohistochemical analysis was used to assess the extravasation of blood-borne immunoglobulin G (IgG) into the brain, an endogenous marker of BBB breakdown, as previously described [34]. Briefly, mounted PFA-fixed brain sections were washed with potassium phosphate-buffered saline (KPBS) (Sigma-Aldrich, MO, USA) and then incubated for 45 min in a permeabilization/blocking solution containing 4% normal goat serum (NGS), 1% BSA (Sigma-Aldrich), and 0.4% Triton X-100 (Sigma-Aldrich) in KPBS. Next, sections were incubated with a biotinylated anti-mouse IgG antibody (Santa Cruz Biotechnology, TX, USA) overnight at 4 °C. The signal was revealed using avidin peroxidase kit (Vectastain Elite; Vector Labs, CA, USA) by immersing the sections for 1 h in avidin-biotin complex (ABC) mixture. Brain sections were washed, stained in 2.5% diaminobenzidine (DAB) solution for 10 min, rinsed and finally mounted onto slides, dried, dehydrated, and cover-slipped with distyrene plasticizer xylene (DPX; Electron Microscopy Sciences, PA, USA). Brain sections were digitized and analyzed for areas exhibiting IgG extravasation using FIJI software. IgG extravasation was represented as percent of fold change of the contralateral as follows; *((contralateral - intact ipsilateral)/contralateral) x 100.* The atrophy size was represented as percent of fold change of the contralateral as follows; *1 - (ipsilateral/contralateral) x 100*.

### Fluoro-Jade B staining

Fluoro-Jade B (FJB) staining was used to assess neuronal degeneration, as previously described [35]. Briefly, free-floating PFA-fixed brain sections were washed with KPBS 3x for 10 min, then mounted onto Superfrost^®^ Plus slides and dried overnight at RT. Mounted sections were fixed with 4% PFA for 45 min, then rinsed 2x with KPBS for 5 min and processed through a cycle of dehydration/rehydration in ethanol (EtOH) (3 min in 50%, 1 min in 70%, 3 min in 100%, 1 min in 70%, 1 min in 50% and 1 min in distilled water). Mounted sections were next treated for 10 min with 0.06% potassium permanganate (MP Biomedicals, CA, USA), rinsed for 1 min with distilled water, and then incubated in 0.2% FJB solution (EMD Millipore, ON, Canada) containing 0.1% acetic acid + 0.0002% 4′,6-diamidino-2-phenylindole (DAPI) in milliQ water for 10 min. Sections were rinsed in milliQ water and dried overnight, then immersed 3x for 2 min in xylene and cover-slipped with DPX. Density of FJB^+^ cells was quantified using unbiased computer-assisted stereological software (Stereologer; SRC Biosciences, FL, USA).

### Immunofluorescence analysis

Free-floating PFA-fixed brain sections were rinsed 3x with KPBS and incubated at RT in a permeabilization/blocking solution containing 4% NGS, 1% BSA, and 0.4% Triton X-100 in KPBS for 45 min. Sections were then incubated with primary antibodies diluted in the permeabilization/blocking solution at 4 °C. The following primary antibodies were used; rat anti-cluster of differentiation (CD31) (1/500; BD Biosciences, ON, Canada; 550,274), rat anti-CD45 (1/500; BD Biosciences; 553076), goat anti-aminopeptidase N (CD13) (1/250; R&D systems; AF2335), goat anti-intercellular adhesion molecule (ICAM)1 (1/250; R&D systems; AF796), rat anti-glial fibrillary acidic protein (GFAP) (1/500; Invitrogen, MA, USA; 13–0300), rabbit anti-aquaporin (AQP)4 (1/250; Invitrogen; PA5-77716), rabbit anti-ionized calcium binding adaptor molecule (IBA1) (1/500; WAKO, ON, Canada; 019-19741), rabbit anti-PDGFRβ (1/250; Abcam, ON, Canada; ab32570), goat anti-collagen IV (1/500; Millipore; AB769), rabbit anti-fibronectin (1/100; Abcam; ab2413), rabbit anti-doublecortin (DCX) (1/500; Abcam; ab18723), mouse anti-tubulin-β III (TUJ1) (1/250; Abcam; ab78078), rabbit anti-claudin 5 (1/250; Abcam; ab15106), and rabbit anti-PDGFRα (1/200; Abcam; ab203491). The next day, sections were rinsed 3x with KPBS, and incubated for 2 h at RT in the dark with one the following secondary antibodies at a dilution of 1/1000; Alexa Fluor 488 donkey anti-rat antibody (Invitrogen; A21208), Cy3 goat anti-mouse (Jackson ImmunoResearch Laboratories Inc, PA, USA; 111-165-003), Cy5 goat anti-rabbit (Invitrogen; A10523), Alexa Fluor 647 donkey anti-goat (Jackson ImmunoResearch Laboratories Inc; 705-605-003), Cy5 goat anti-rat (Jackson ImmunoResearch Laboratories Inc; 112-175-143). Brain sections were then rinsed 2x with KPBS, incubated with DAPI (1/10 000; Invitrogen) for 5 min, mounted onto Superfrost^®^ Plus slides, and cover-slipped with Fluoromount-G^®^ anti-fade medium (Electron Microscopy Sciences). Epifluorescence images were acquired using Axio Observer microscope equipped with a module for optical sectioning (Apotome.2) and Axiocam 503 monochrome camera, and then processed using ZEN Imaging Software (Carl Zeiss Canada, ON, Canada). The density of DCX^+^ cells and TUJ1^+^ cells were assessed using unbiased computer-assisted stereological software (Stereologer; SRC Biosciences), as described [36]. Density of the microvessels in 3 regions of interest (ROIs; 300 µm^2^) taken with a 20X objective in the perilesional and intralesional sites were measured using FIJI software, as described [37].

### RNAscope™ fluorescent in situ hybridization (F.I.S.H)

Free-floating brain sections were mounted onto Superfrost^®^ Plus slide and kept at -20 °C to dry for 1 h before being baked for 30 min at 60 °C. The mounted sections were fixed for 15 min in 4% PFA and washed with 0.1 M PBS at 4 °C. Mounted sections were immediately dehydrated using ethanol and incubated in hydrogen peroxide (H_2_O_2_) for 10 min. Following 10 min of retrieval, mounted sections were immersed in a 100% ethanol bath and a hydrophobic barrier around the sections was drawn. RNAscope^®^ fluorescent multiplex reagent kit was used following manufacturer’s recommendations (Advanced Cell Diagnostics, CA, USA). Five drops of Protease III cocktail were added to completely cover each section, followed by an incubation at RT for 40 min. Following series of washes with milliQ water, 4 drops of mouse RNAscope^®^ Probe-*PDGFRβ*-C1 (411381), RNAscope^®^ Probe-*PDGF-D*-C1 (424311), RNAscope^®^ Probe-*IGF1*-C3 (443901-C3), and RNAscope^®^ Probe-*PECAM1*-C3 (316721-C3) were added to entirely cover each section, and slides were placed into a slide rack, and inserted in the humidity control tray of HybEZ™ Oven for 2 h at 40 °C. The humidity control tray was removed from the HybEZ™ Oven, one slide at a time, and excess of liquid was removed and washed with 1X wash buffer. Four drops of Amp-1 were added to entirely cover each brain section, which were incubated again in the HybEZ™ Oven for 30 min at 40 °C. This process was repeated with Amp-2 for 30 min and Amp-3 for 15 min. *PDGF-D* and *PECAM1* mRNA transcripts were simultaneously detected using Opal™ 690 (1/700; AKOYA Biosciences^®^, MA, USA; OP-001006) and Opal™ 570 (1/700; OP-001003), respectively. *PDGFRβ* and *IGF1* mRNA transcripts were simultaneously detected using Opal™ 690 (1/700) and Opal™ 570 (1/700), respectively. Finally, 4 drops of DAPI (provided in the kit) were added to each brain section and kept for 30 s, followed by series of washes with 1X wash buffer. Slides were next mounted with 110 µL of Fluoromount-G^®^ anti-fade medium, carefully cover-slipped, and placed at 4 °C in the dark until analysis of fluorescence intensity under Axio Observer microscope (Carl Zeiss, Canada).

### Neurobehavioral analysis

The rotarod test was used to evaluate the motor coordination of mice [38]. The apparatus consisted of a black striated rod (diameter: 3 cm) separated into five individual compartments (width: 5 cm), 20 cm high from 5 tilting planks (Harvard Apparatus^®^; LE8200) [39]. The animals were placed on a rotating rod at 4 rpm and speed progressively increases to maximum 40 rpm for a total of 120 s in each test. The latency to fall from the rotating rod was recorded (maximum time: 120 s). Animals were trained prior to MCAo during 3 consecutive days, followed by a baseline test at day 4 [40]. Upon MCAo, animals were assessed at 24 h, 1 and 2 weeks to assess the recovery of motor coordination.

The pole test was used to assess the overall locomotor function after ischemic stroke. Briefly, each animal was separately placed on the top of a 60 cm vertical pole with a rough surface which has a diameter of 10 mm. Time taken to reach the ground was recorded. In case the animal stopped during a run, then the trial was excluded and repeated [41]. In case the animal was unsuccessful in performing the task by falling or sliding, a score of 20 s was accorded, which was considered as the maximum time. Each animal was tested prior to MCAo and all animals were afterwards randomly assigned to an experimental group. Animals were tested 24 h, 1 and 2 weeks after MCAo. For each time point, each animal was tested 3 times and the average was used for statistical analysis.

### Imaging and analysis of brain perfusion

Laser Speckle contrast imaging (LSCI) was used to investigate longitudinal brain perfusion, which reflects regional CBF changes, as previously described [42]. Prior to the procedure, mouse head was shaved, lidocaine/bupivacaine solution applied on the incision site (100 µL) and the ears (50 µL/ear), and the skin was disinfected. Mice were placed on a stereotaxic frame (RWD Life Science Inc., CA, USA) and anaesthetized using 2% isoflurane (95% O_2_, 2L/min). The skull was then exposed by removing the skin using fine-tip forceps, and CBF was visualized using 2D laser Speckle blood flow imager (OMEGAZONE OZ-3; Omegawave Inc.). OZ-3 is equipped with a visible and near infra-red (NIR) CCD camera, which allows showing real images continuously, and to compare the color difference between real and blood flow images. Continuous colored blood flow images were recorded at high resolution (HR) mode using LSI measurement software and analyzed using LIA software that enables simultaneous quantification of relative blood perfusion in different ROIs. The relative brain perfusion in each hemisphere was calculated by averaging (20 consecutive raw Speckle images in each ROI), and the ratio of blood perfusion in the ipsilateral over contralateral hemisphere was calculated. Blood perfusion was measured 5 min (immediately after occlusion onset to establish a post-stroke baseline), 24 h and 1 week after MCAo. The changes in brain perfusion were measured in the same animals 5 min (time 0), 24 h (time 1) and 1 week (time 7) after ischemic stroke. The temporal changes between time 0–1 (acute changes immediately upon PDGF-D administration), and time 0–7 (delayed changes upon reportative PDGF-D administration) were examined by fitting a linear ordinary least squares (OLS) model using the ‘lm’ function in the R statistical software. The model formula (response ~ time*group) included the interaction between days after ischemic stroke and treatment groups. Plots were generated using the ‘interact_plot’ function from the interactions package and show the mean slope [a slope = 24 h interval] with 95% confidence intervals (CI). The codes used to run the changes in bain perfusion are available online: 10.5281/zenodo.10839501.

### Isolation of brain microvessels

Mice were euthanized via a transcardiac perfusion with 0.9% NaCl. Brains were retrieved and were transferred into PBS medium on ice. After cerebellum removal, the ipsilateral and contralateral hemispheres were separated, and the MCA overlaying regions were dissected. Brain samples from each hemisphere were homogenized in a microvessel isolation buffer (MIB) (15 mM NaCl, 4 mM KCl, 3 mM CaCl2 and 12 mM MgCl2). Homogenates were centrifuged at 900xg for 10 min at 4 °C. Supernatants were removed and pellets were resuspended with 2 mL of dextran 30% into MIB. Samples were centrifuged again at 3600xg for 20 min at 4 °C. Supernatants were removed and pellets were resuspended with 2 mL of MIB. Samples were then filtered through a 30 μm filter (EMD Millipore; NY3002500) to trap microvessels. The isolated microvessels were lysated using 200 µL NP40 lysis buffer supplemented with 1% protease inhibitor cocktail (Sigma-Aldrich) and 1% phosphatase inhibitor cocktail (Sigma-Aldrich) and stored at -20 °C for Western blot analysis.

### Cell culture

Primary human brain vascular pericytes (HBVP) (ScienCell Research Laboratories, CA, USA; 1200) were used to investigate the molecular mechanisms underlying PDGF-D action. Moreover, immortalized human brain endothelial cells (iHBEC) (Cedarlane Laboratories, ON, Canada; CRL-3245) displaying major BBB features [43, 44] were used to assess pericyte-endothelial cell interaction. HBVP were cultured at 37 °C in 5% CO_2_, 95% air in a Dulbecco’s modified Eagle’s medium (DMEM) glucose-normal medium (Multicell; Wisent, QC, Canada) containing 2% fetal bovine serum (FBS), 100 U/mL pericytes growth serum (PGS) and 100 U/mL streptomycin/penicillin. For iHBEC, the surface of flasks/wells was pre-coated with 0.1% gelatin diluted in water (STEMCELL Technologies, BC, Canada) for at least 1 h before iHBEC were seeded. iHBEC were cultured at 37 °C in 5% CO_2_, 95% air in endothelial cell medium (ECM; ScienCell) containing 2% FBS, 100 U/mL endothelial cell growth serum (ECGS), and 100 U/mL streptomycin/penicillin. In all experiments, cells were grown to 80% confluence and subjected to a maximum of 7 passages. Cells were treated with saline, 40 or 80 ng/mL of PDGF-D (R&D Systems). At the end of each experiment, cell culture medium was collected, and cells were harvested for protein extraction for further analysis.

### Oxygen and glucose deprivation

To investigate the response of pericytes to ischemia and reperfusion-like conditions, cells were incubated in oxygen and glucose deprived (OGD) condition, as previously described [12, 37]. HBVP were seeded at 3 × 10^5^ cells/well, in a 12-well plate (Corning, NY, USA). OGD was induced by incubating cells at 37 °C in DMEM-glucose free medium (Multicell; Wisent) under hypoxic condition (1%O_2_, 5%CO_2_) overnight for 18 h using a hypoxia chamber (STEMCELL Technologies) to generate ischemia-like conditions. Next day, DMEM-glucose free medium was immediately replaced by DMEM glucose-normal medium, and cells were incubated under normal oxygenation conditions, mimicking the effect of reperfusion, with or without rh-PDGF-D at 80 ng/mL for 24 h. As control, cells were incubated at 37 °C in DMEM glucose-normal medium (MultiCell; Wisent) under normal oxygen conditions (normoxia). Cells were harvested to extract proteins for further molecular analysis.

### Western blot analysis

HBVP were lysated similarly to isolated microvessels using NP40 lysis buffer supplemented with 1% protease inhibitor cocktail (Sigma-Aldrich) and 1% phosphatase inhibitor cocktail (Sigma-Aldrich) [45]. Total protein concentration was determined in each of the samples using bicinchoninic acid (BCA) (QuantiPro Assay Kit; Sigma-Aldrich) [45]. Protein samples (20 µg) were mixed with 2X sodium dodecyl sulfate (SDS)-loading buffer and heated for 10 min at 95 °C. Samples were run on 8% or 12% polyacrylamide gel electrophoresis (SDS-PAGE) and subjected to electrophoresis using Mini-PROTEAN^®^ Tetra Cell (Bio-Rad, Hercules, CA, USA). After migration, resolved protein bands were transferred onto a 0.45 μm polyvinylidene fluoride (PVDF) membrane (EMD Millipore) for 75 min at 100 V on ice using Mini Trans-Blot^®^ Electrophoretic Transfer Cell (Bio-Rad). The PVDF membranes were rinsed 3x for 10 min with 0.1 M TBS solution containing 0.5% Tween-20 (TBS-T; Sigma-Aldrich) and blocked in TBS-T 5% (w/v) skim milk for 30 min at RT. The PVDF membranes were then incubated overnight at 4 °C with the following primary antibodies diluted at 1/1000 in TBS-T solution; rabbit anti-NOTCH3 (Abcam; ab23426), rabbit anti-BCL2 (Cell Signaling Technology, MA, USA; 2876S), rabbit anti-PDGF-D (Abcam; ab181845), and mouse anti-actin (EMD Millipore; MAB1501). Primary antibodies were detected with the appropriate horseradish peroxidase (HRP)-conjugated secondary antibodies (Jackson ImmunoResearch Laboratories Inc.) that were diluted at 1/10 000 in TBS-T and revealed by enhanced chemiluminescence plus (ECL) solution (Bio-Rad). Actin was used to ensure equal protein loading. Blots were digitized using Biorad chemidoc XRS^+^ system (Bio-Rad). Digitized blots were densitometrically analyzed with FIJI software corrected for protein loading by means of actin, and expressed as relative values comparing different groups, as described [37].

### Cell migration assay

The wound healing assay was used to assess the migratory capacity of HBVP. Briefly, 3 × 10^5^ HBVP per well (3 replicates per condition) were plated into a 12-well plate and incubated under the appropriate condition to reach confluence. The cell monolayer was scratched at well midline using a 1 mm pipette tip and washed with serum-free medium to remove detached cells. HBVP were then cultured in complete medium supplemented with vehicle or rh-PDGF-D (80 ng/mL) under normoxic or OGD conditions. Brightfield images were acquired using the 10X objective of Axio Observer microscope (Carl Zeiss, Canada) immediately after scratching cells (0 h) and 24 h later. Cell migration was assessed by quantifying wound closure, calculated as follows; *(A*_*0*_*- A*_*n*_*)/A*_*0*_* × 100*, where A_0_ represents initial complete area of the wound, and A_n_ represents the remaining open area of the wound.

### Proteome profiler™ human angiogenesis array

Proteome Profiler™ human angiogenesis array kit (R&D Systems; ARY007) was used to simultaneously profile the expression of 55 angiogenesis-related proteins of unstimulated and PDGF-D-stimulated HBVP under normoxic or OGD conditions at 24 h, following manufacturer’s instructions. Briefly, the membranes were blocked with 2 mL of array buffer 5 for 1 h on an orbital shaker. Meanwhile, the sample/antibody mixture (170 µL of samples, 500 µL of array buffer 4, 830 µL of array buffer 5, 15 µL detection antibody cocktail) was prepared and incubated for 1 h at RT. Afterwards, the array buffer 5 was removed from the membrane and replaced with the sample/antibody mixture which was incubated at 4 °C overnight on a shaker. The next day, each membrane was washed 3x for 10 min with a 1X wash buffer and then incubated with Streptavidin-HRP for 30 min at RT while shaking. Membranes were washed 3x for 10 min in 1X wash buffer and were incubated with 1 mL of Chemi Reagent Mix for 5 min and digitized using Biorad Chemidoc XRS^+^ (Biorad). Digitized blots were densitometrically analyzed with FIJI software and expressed as relative values comparing PDGF-D-treated with non-treated cells under normoxic or OGD conditions.

### In vitro tube formation assay

The in vitro tube formation assay (ScienCell Research Laboratories) was used to assess the capacity of endothelial cells cultured on extracellular matrix (ECM) to form of 3-dimensional tubular structure. Briefly, a ready to use ECM solution (ScienCell Research Laboratories) was added into each well of a 96-well plate and incubated for 1 h at 37 °C in 5% CO_2_, 95% air. In a first series of experiments, 0.5 × 10^5^ iHBEC were either, (1) seeded alone without treatment, (2) treated with rh-PDGF-D or (3) with the conditioned medium (CM) of OGD-exposed HBVP stimulated with rh-PDGF-D. In a second series of experiments, 0.5 × 10^5^ iHBEC were co-cultured with 0.5 × 10^4^ HBVP and the co-culture was either, (1) maintained without treatment, (2) treated with rh-PDGF-D or (3) with the CM of OGD-exposed HBVP stimulated with rh-PDGF-D. Brightfield images were acquired using the 10X objective of Axio Observer microscope (Carl Zeiss, Canada) of tube-like structures 8 h later.

### Statistical analysis

Results are presented as boxplot with min/max or line chart ± SD. Data distribution normality was assessed using the Shapiro Wilk test. For comparisons between two groups, unpaired two-tailed t-test were used. For multiple comparison, one-way or two-way analysis of variance (ANOVA) followed by Tukey’s post-hoc test was used. For data not passing the normality test, Mann-Whitney test or Kruskal-Wallis followed by Dunn’s multiple comparisons test were used. *P* < 0.05 was considered statistically significant. Statistical analyses were carried out using GraphPad Prism Version 9.0 for OS X (GraphPad Software, CA, USA). For brain perfusion analysis, R statistical software was used and R-Squared (R^2^) for linear regression and 95% CI for regression interception were calculated. Higher R^2^ values suggest a greater difference and 95% CI outlines the relative extent of brain perfusion changes.

## Results

### PDGF-D is predominantly expressed in brain endothelial cells and its expression is transiently induced after ischemic stroke

**Fig. 1:**
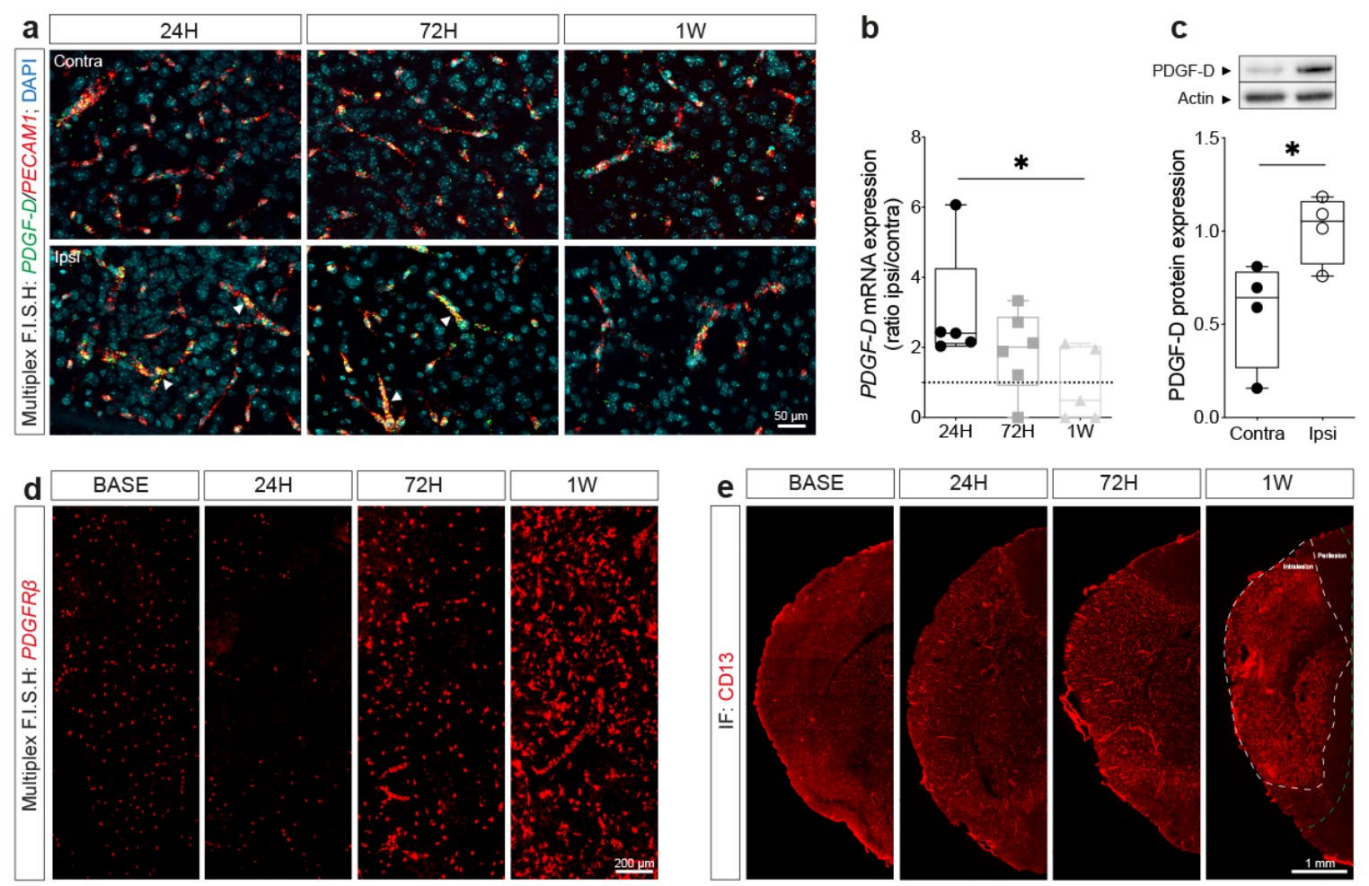
Endothelial PDGF-D expression is transiently induced after ischemic stroke. (**a**) Representative multiplex RNAscope^®^ F.I.S.H images showing *PDGF-D* and *PECAM1* relative mRNA expression in the contralateral and ipsilateral hemispheres at 24, 72 h and 1 week after ischemic stroke (arrowheads underpin PDGF-D expression in vascular structures). (**b**) Analysis of *PDGF-D* mRNA temporal expression at 24, 72 h and 1 week after stroke, shown as ipsilateral/contralateral ratio. (**c**) Western blot analysis showing PDGF-D expression in isolated brain microvessels in contralateral and ipsilateral hemispheres 24 h after ischemic stroke. (**d**) Representative multiplex RNAscope^®^ F.I.S.H images showing *PDGFRβ* mRNA expression in the ipsilateral hemispheres at 24, 72 h and 1 week after ischemic stroke. (**e**) Representative fluorescence images of CD13 immunolabeling in the ipsilateral hemispheres at 24, 72 h and 1 week after ischemic stroke (the intralesional region is associated with dense CD13 reactivity surrounded by the perilesional region). Data are boxplot with min/max (**b**) (*n* = 5–6 animals/groups), (**c**) (*n* = 0.4 animals/group). ^*^P < 0.05 (**b**) 1 week compared to 24 h (Kruskal-Wallis/Dunn’s test) (**c**) or contralateral compared ipsilateral at 24 h (unpaired two-tailed t-test). BASE, baseline; H, hour; W, week; Contra, contralateral; Ipsi, ipsilateral

In the adult brain, PDGF-D was reported to be mainly expressed in vascular cells [29]. PDGF-D brain expression is minimal under physiological conditions, whereas its expression increases under pathological conditions [46]. PDGF-D expression pattern in the brain after ischemic stroke remains unexplored. We have first analyzed the simultaneous spatiotemporal expression of *PDGF-D* and *PECAM1* mRNA using multiplex RNAscope^®^ F.I.S.H **(**Fig. 1a**)**. Interestingly, we found that *PDGF-D* mRNA transcripts (puncta) were potently induced (~ 2-fold) in endothelial cells expressing *PECAM1* mRNA transcripts at the lesion site 24 h after ischemic stroke **(**Fig. 1b**)**. *PDGF-D* mRNA expression progressively decreased over time, beginning at 72 h, to reach pre-stroke basal levels at 1 week (24 H vs. 1 W, *P* = 0.0348) **(**Fig. 1b**)**. To further validate the endothelial source of PDGF-D, brain microvessels were isolated 24 h after ischemic stroke, a time point corresponding to mRNA expression peak **(**Fig. 1c**)**. Our analysis showed that PDGF-D protein expression was potently induced specifically in endothelial cells isolated from the ipsilateral compared to the contralateral hemisphere (*P* = 0.0381). We evaluated next pericyte reactivity concomitantly with PDGF-D regulation. Although PDGFRβ protein expression was reportedly shown to be induced after ischemic stroke, little is known about its spatiotemporal transcriptomic regulation. Using multiplex RNAscope^®^ F.I.S.H, we showed that *PDGFRβ* mRNA expression was slightly reduced at the lesion site 24 h after ischemic stroke followed by an increased expression that peaked at 1 week (Fig. 1d). The immunolabeling of CD13, a pericyte marker that is not expressed in other brain perivascular cells, including fibroblasts, outlined the temporal activation of pericytes and subsequent recruitment to the intralesional site at 1 week after ischemic stroke (Fig. 1e). These findings indicate that PDGF-D expression is transiently and specifically induced in brain endothelial cells in the sub-acute phase of stroke.

### Subacute PDGF-D specific downregulation exacerbates neuronal and vascular damage

**Fig. 2:**
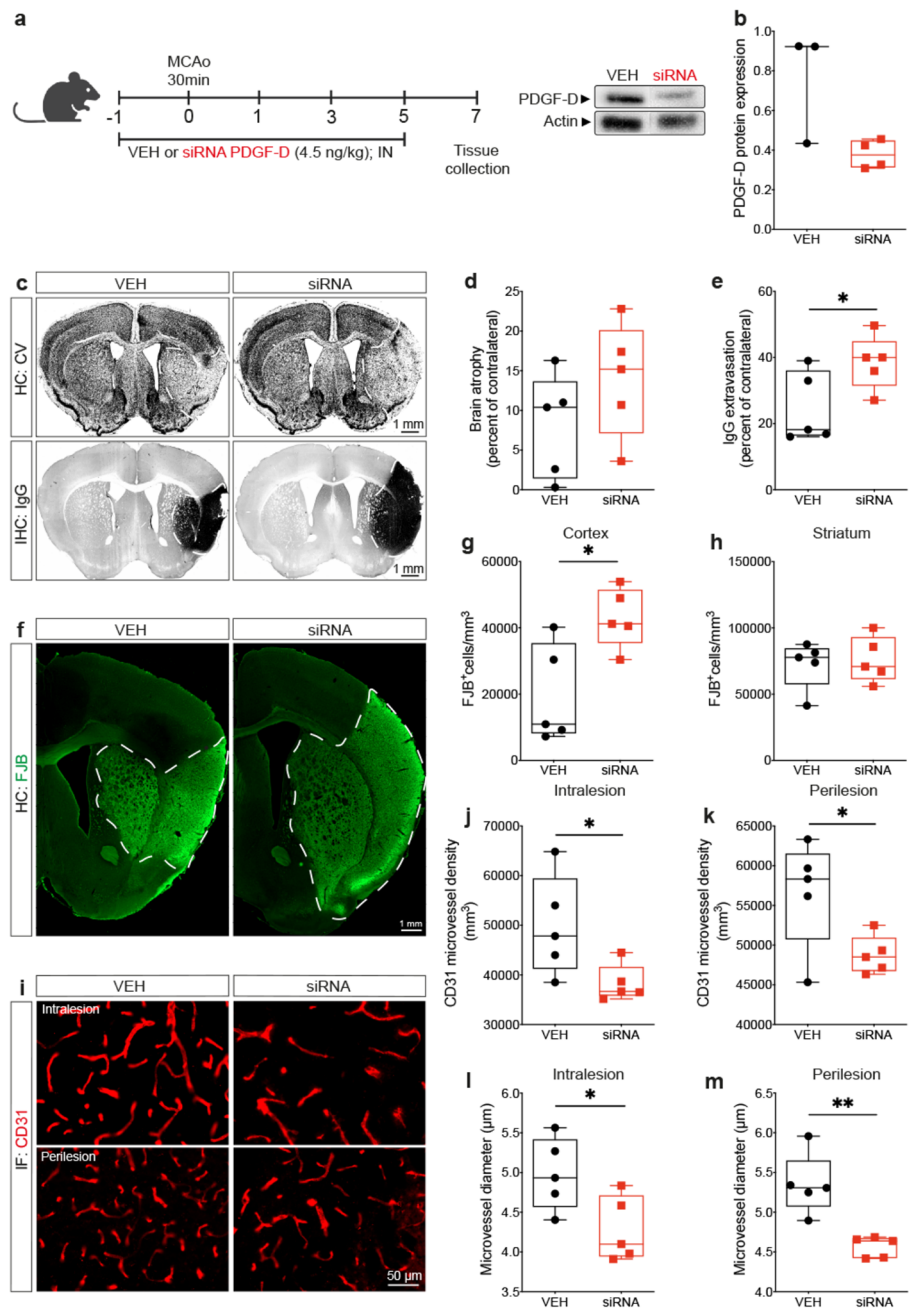
Subacute PDGF-D brain downregulation aggravates neuronal and microvascular damage after stroke. (**a**) Schematic illustration of the experimental design of *PDGF-D* siRNA intranasal delivery 24 h prior to ischemic stroke and repeated every 2^nd^ day until 1 week. (**b**) Western blot analysis showing PDGF-D expression in the brain of vehicle (VEH)- and siRNA-treated mice, 2 days after intranasal administration. (**c**) Representative images of cresyl violet histology showing the injury area surrounded by a white dashed line in the ipsilateral hemisphere, and IgG immunohistochemistry showing IgG extravasation area in black surrounded by a white dashed line in the ipsilateral hemisphere, 1 week after stroke. (**d**) Analysis of brain atrophy represented as percent of contralateral in VEH- and siRNA-treated mice, 1 week after stroke. (**e**) Analysis of IgG extravasation represented as percent of contralateral in VEH- and siRNA-treated mice, 1 week after stroke. (**f**) Representative fluorescence images of FJB^+^ degenerating neurons surrounded by a white dashed in the ipsilateral cortex and striatum, 1 week after stroke. Stereological analysis of the density of FJB^+^ degenerating neurons in the ipsilateral (**g**) cortex and (**h**) striatum, 1 week after stroke. (**i**) Representative fluorescence images of CD31 immunolabeling in the intralesional and perilesional sites in VEH- and siRNA-treated mice, 1 week after stroke. Analysis of the density of CD31^+^ microvessels in the (**j**) intralesional and **(k)** perilesional sites, 1 week after stroke. Analysis of the average diameter of CD31^+^ microvessels in the **(l)** intralesional and (**m**) perilesional sites, 1 week after stroke. Data are boxplot with min/max (**b**) (*n* = 3–4 animals/groups), (**c-m**) (*n* = 5 animals/groups). ^*^P < 0.05/^**^P < 0.01 compared to siRNA-treated mice (Mann-Whitney test or unpaired two-tailed t-test). IN, intranasal; HC, histochemistry; IHC, immunohistochemistry; IF, immunofluorescence.

Previous reports have indicated that PDGF-D depletion causes mild vascular impairments in absence of pathological conditions [29]. Herein, we aimed to analyze the biological significance of PDGF-D transient subacute induction after ischemic stroke. For this purpose, PDGF-D expression was downregulated through siRNA delivery into the brain via the intranasal route, 24 h prior to stroke, and repeated every 2^nd^ day, followed by euthanization of mice 1 week after ischemic stroke onset **(**Fig. 2a**)**. This strategy was chosen to enable a stable downregulation of PDGF-D throughout the subacute phase during which its expression was induced, as siRNA silencing effects peak 2 days post-transfection [47]. Western blot analysis revealed that siRNA delivery was associated with a downregulation of PDGF-D protein expression in the brain of mice (VEH:0.9232 ± 0.4942 vs. siRNA:0.3759 ± 0.1342) **(**Fig. 2b**)**. First, cresyl violet histological and IgG immunohistological analyses were used to assess structural damage, namely brain atrophy and extravasation of blood-borne IgG across the BBB, respectively (Fig. 2c). Attenuation of PDGF-D post-stroke induction did not affect brain atrophy (VEH:8.120 ± 6.558, siRNA:13.94 ± 7.237) (Fig. 2d), whereas it aggravated IgG infiltration into the injured brain 1 week after stroke (*P* = 0.0489) (Fig. 2e), outlining an increased vascular permeability. Next, we assessed the impact of PDGF-D downregulation on cellular damage, namely neuronal degeneration, 1 week after ischemic stroke using FJB staining (Fig. 2f). Our analysis showed that attenuating PDGF-D subacute expression increased the density of FJB^+^ cells in the cortex 1 week after stroke (*P* = 0.0165) (Fig. 2g), translating an exacerbation of neuronal degeneration, which could not be detected in the striatum (*P* = 0.7576) (Fig. 2h). This suggests that neuronal degeneration upon PDGF-D downregulation was more potent in densely vascularized structures, such as cortex. Ischemic stroke is associated with activation of different endogenous pro-angiogenic responses, as an attempt to improve injured tissue vascularization [11, 12]. As PDGF ligands play critical roles in vascular remodeling [48], we evaluated the impact of PDGF-D subacute downregulation on vascular density and morphology after ischemic stroke by immunolabeling CD31 (Fig. 2i). Our analysis showed that attenuation of PDGF-D subacute expression decreased the density of CD31^+^ microvessels in the intralesional (*P* = 0.0434) (Fig. 2j) as well as the perilesional (*P* = 0.0418) (Fig. 2k) sites, 1 week after stroke. The reduced microvascular density was associated with a diminution of the average diameter of CD31^+^ microvessels in the intralesional (*P* = 0.0331) (Fig. 2l) and the perilesional (*P* = 0.0025) (Fig. 2m) sites, outlining a deregulation of vascular structure translated by exacerbation of post-stroke stalling, constriction, and reduced patency. These findings suggest that PDGF-D induction after stroke contributes to the maintenance of vascular integrity and subsequent attenuation of neuronal loss.

### Increased PDGF-D subacute bioavailability alleviates neuronal loss and vascular injury

**Fig. 3:**
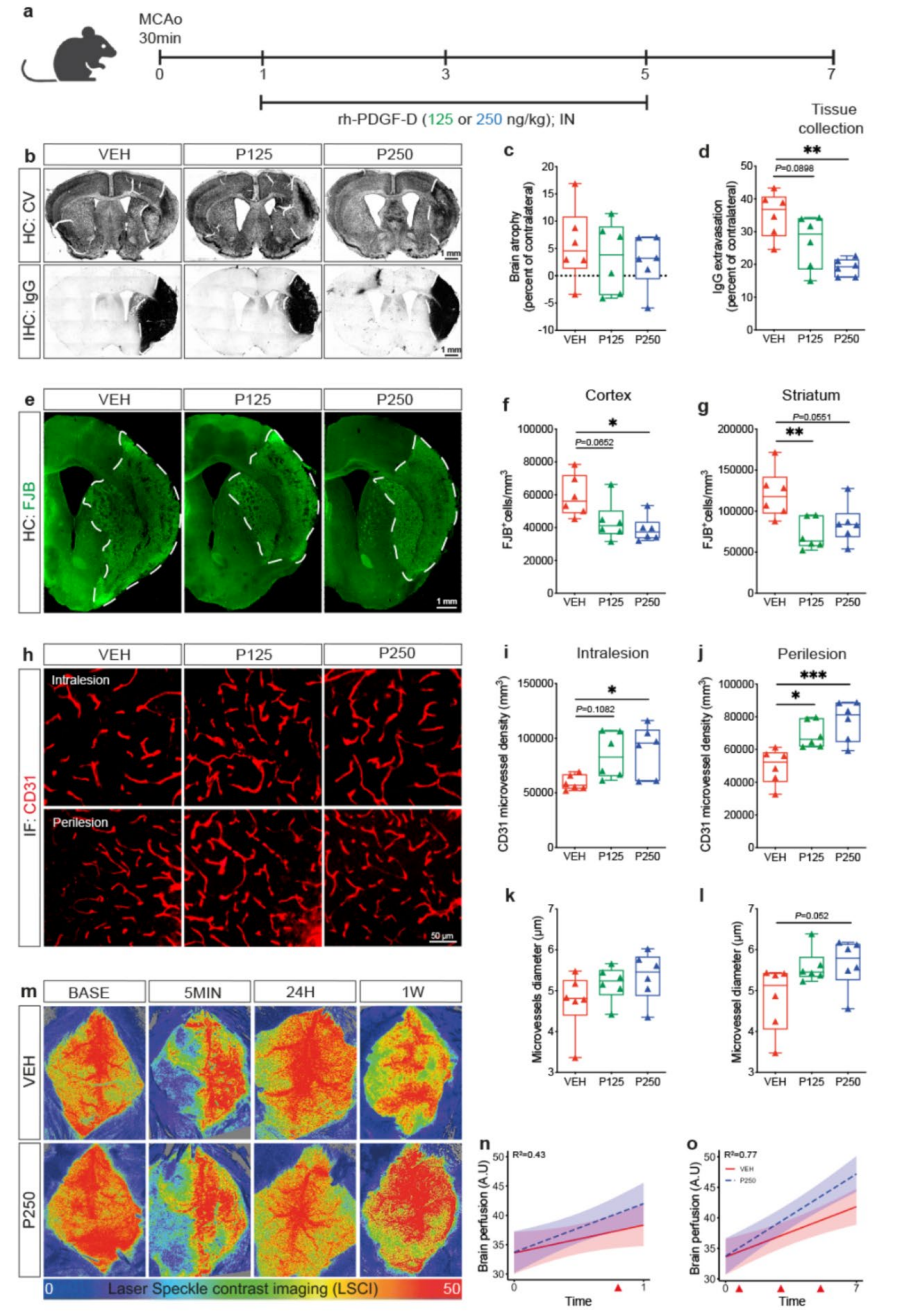
Increased PDGF-D subacute abundance attenuates neuronal loss and rescues microvascular structure after stroke. (**a**) Schematic illustration of the experimental design of rh-PDGF-D intranasal administration 24 h after ischemic stroke and repeated every 2^nd^ day until 1 week after stroke. (**b**) Representative images of cresyl violet histology showing the injury area surrounded by a dashed line in the ipsilateral hemisphere and IgG immunohistochemistry showing IgG extravasation in black surrounded by a white dashed line in the ipsilateral hemisphere, 1 week after stroke. (**c**) Analysis of brain atrophy represented as percent of contralateral in vehicle (VEH)-, rh-PDGF-D 125 ng/kg (P125)-, or rh-PDGF-D 250 ng/kg (P250)-treated animals, 1 week after stroke. (**d**) Analysis of IgG extravasation represented as percent of contralateral in VEH, P125 or P250 groups, 1 week after ischemic stroke. (**e**) Representative fluorescence images of FJB^+^ degenerating neurons surrounded by white dashed line in the ipsilateral cortex and striatum, 1 week after ischemic stroke. Stereological analysis of the density of FJB^+^ degenerating neurons in the ipsilateral (**f**) cortex and (**g**) striatum, 1 week after ischemic stroke. (**h**) Representative fluorescence images of CD31 immunolabeling in the intralesional and perilesional sites in VEH, P125, or P250 groups, 1 week after ischemic stroke. Analysis of the density of CD31^+^ microvessels in the (**i**) intralesional and (**j**) perilesional site, 1 week after ischemic stroke. Analysis of the average diameter of CD31^+^ microvessels in the (**k**) intralesional and (**l**) perilesional site, 1 week after stroke. (**m**) Representative brain perfusion images acquired using LSCI at baseline, 5 min, 24 h and 1 week after ischemic stroke in VEH and P250 groups. (**n**) Linear regression analysis of brain perfusion changes using R statistical software between time 0 (5 min post-occlusion) and time 1 (24 h post-occlusion) or (**o**) time 7 (1 week post-occlusion) (red arrowheads indicate the time points at which PDGF-D was delivered into the brain after ischemic stroke). Data are boxplot with min/max (**a-l**) (*n* = 6 animals/group), (**m, o**) (*n* = 5 animals/group). ^*^P < 0.05/^**^P < 0.01/^***^P < 0.001 compared to P125 or P250 groups (one-way ANOVA/Tukey’s test; **(l)** Kruskal-Wallis/Dunn’s test; **(n, o)** Linear regression using R statistical software). IN, intranasal; HC, histochemistry; IHC, immunohistochemistry; IF, immunofluorescence; BASE, baseline; MIN, minute; H, hour; W, week

Our hereabove findings showed that attenuation of PDGF-D subacute induction compromises neuronal damage and vascular integrity. Therefore, we aimed next to evaluate the impact of increasing subacute PDGF-D bioavailability on structural outcomes. For this purpose, active rh-PDGF-D was non-invasively delivered into the brain through the intranasal route, 24 h after ischemic stroke, and repeated every 2^nd^ day until euthanization of mice at 1 week (Fig. 3a). This strategy was chosen to maintain elevated levels of active PDGF-D in the brain throughout the subacute phase. Using cresyl violet histology and IgG immunohistology analyses (Fig. 3b), we assessed the impact of increased PDGF-D bioavailability in the subacute phase on structural damage. Our analysis showed that PDGF-D did not affect brain atrophy (VEH:5.717 ± 6.829, P125:3.317 ± 6.496, P250:2.633 ± 7.787) (Fig. 3c), but strongly reduced IgG extravasation (VEH:35.2 ± 6.928, P125:26.91 ± 8.013; VEH vs. P250, *P* = 0.0013) (Fig. 3d) 1 week after ischemic stroke. We next assessed neuronal degeneration using FJB staining (Fig. 3e), and found that PDGF-D dose-dependently reduced the density of FJB^+^ cells in the cortex (VEH vs. P125, *P* = 0.0652; VEH vs. P250; *P* = 0.0153) (Fig. 3f), as well as in the striatum (VEH vs. P125, *P* = 0.0087; VEH vs. P250, *P* = 0.0551) (Fig. 3g), 1 week after stroke. Afterwards, we evaluated tissue vascularization in the ipsilateral hemisphere using CD31 immunolabeling (Fig. 3h), and our analysis showed that PDGF-D dose-dependently increased the density of CD31^+^ microvessels in the intralesional (VEH:551.8 ± 63.32, P125:754.2 ± 166.9, *P =* 0.1082; VEH vs. P250, *P* = 0.0251) (Fig. 3i) as well as the perilesional (VEH vs. P125, *P* = 0.0104; VEH vs. P250, *P* = 0.0005) (Fig. 3j) sites, 1 week after stroke. Furthermore, PDGF-D enhanced bioavailablity increased the average diameter of CD31^+^ microvessels in the intralesional (VEH:4.73 ± 0.7272, P125:5.178 ± 0.4283, P250:5.351 ± 0.5973) (Fig. 3k) as well as the perilesional (VEH:4.806 ± 0.7981, P125:5.588 ± 0.4129, P250:5.657 ± 0.61) (Fig. 3l) sites, 1 week after ischemic stroke. This outlines an alleviated microvascular stalling and constriction, and thus an enhanced patency. Next, we investigated the functional consequences of the improved microvascular density and patency mediated by PDGF-D increased subacute bioavailability on brain hemodynamics using LSCI (Fig. 3m). Our analysis demonstrated that the regression for time 0 (5 min) - time 1 (24 h) after ischemic stroke (R^2^ = 0.43) showed in VEH an intercept of 33.5 (95% CI = 30 -37.1) in brain perfusion with a mean increase of 4.7 (95% CI = -0.32–9.7) 24 h after ischemic stroke, whereas the mean difference in slope in P250 was 3.5 (95% CI = -3.5–10.7) (Fig. 3n). This suggests that brain perfusion began to increase immediately upon PDGF-D brain intranasal infusion. Notably, the regression for time 0 (5 min) - time 7 (1 week) after ischemic stroke (R^2^ = 0.77) yielded in VEH an intercept of 33.5 (95% CI = 30.6–36.5) and mean increase of 8.19 (with a mean slope of 1.17) (95%CI = 0.57–1.76). The slope difference for P250 was 3.56 (95% CI = -0.08–1.59) (Fig. 3o). The more positive CI between time 0 (5 min) and time 7 (1 week) suggests that the differences in brain perfusion between VEH and P250 are more pronounced at 1 week after ischemic stroke. This outlines a progressive improvement of brain perfusion in response to the increased subacute bioavailability of PDGF-D. These findings suggest that PDGF-D induction reduces subacute neuronal loss by promoting functional neovascularization that enables a proper perfusion of the injured tissue.

### PDGF-D promotes association of pericytes with the microvasculature and subsequent stability

**Fig. 4:**
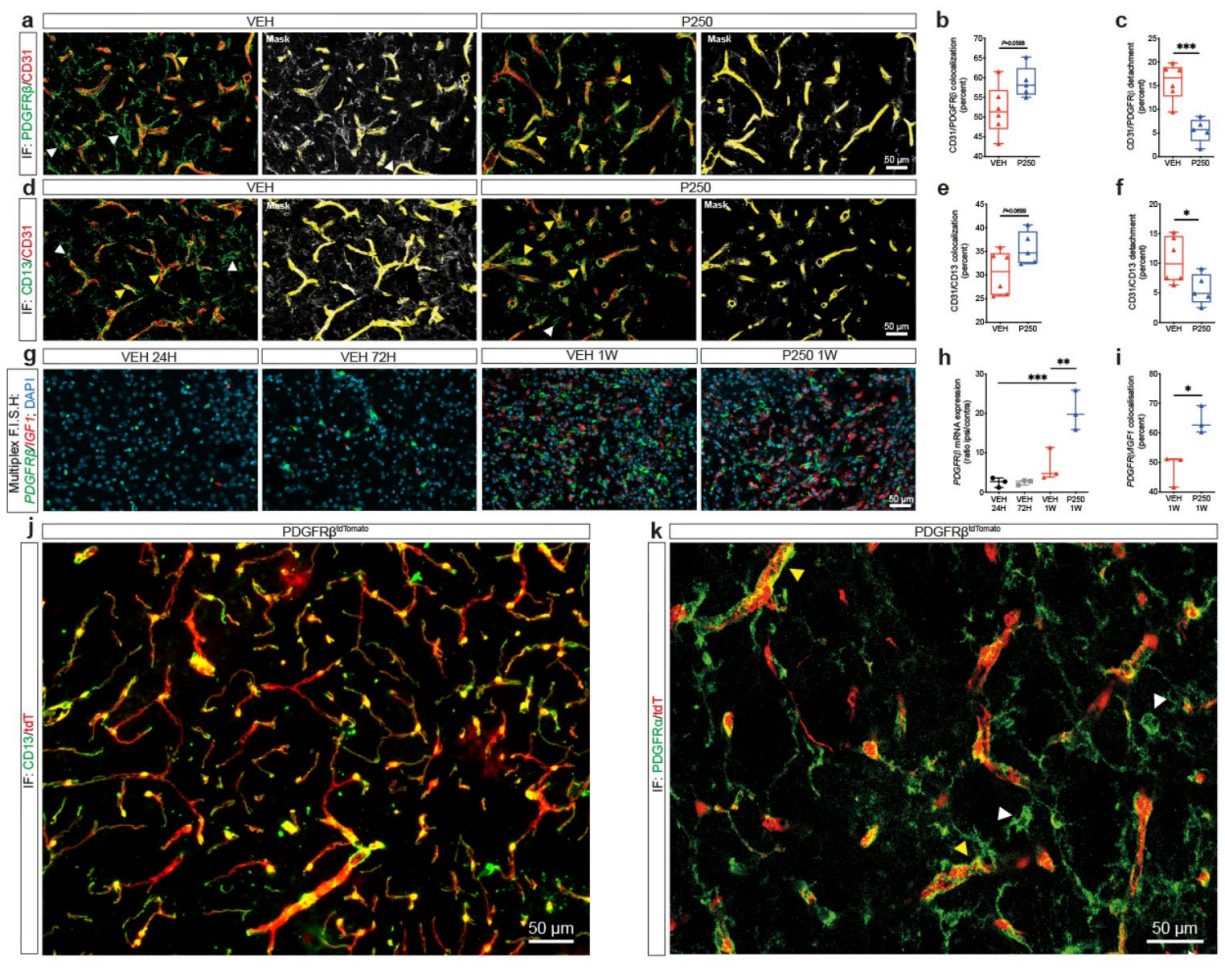
PDGF-D improves subacute pericyte-endothelial cell interaction. (**a**) Representative fluorescence images of PDGFRβ and CD31 co-immunolabeling at the lesion site in vehicle (VEH)- or rh-PDGF-D 250 ng/kg (P250)-treated mice, 1 week after stroke (white arrowheads represent PDGFRβ^+^ cell detachment from CD31^+^ microvessels; yellow arrowheads represent PDGFRβ^+^ cell association with CD31^+^ microvessels). (**b**) Analysis of CD31 microvessels coverage by PDGFRβ^+^ cells and (**c**) PDGFRβ^+^ cells detached from CD31^+^ microvessels at the lesion site, 1 week after stroke. (**d**) Representative fluorescence images of CD13 and CD31 co-immunolabeling at the lesion site in VEH and P250 groups, 1 week after ischemic stroke (white arrowheads represent CD13^+^ cell detachment from CD31^+^ microvessels; yellow arrowheads represent CD13^+^ cell association with CD31^+^ microvessels). (**e**) Analysis of CD31 microvessel coverage by CD13^+^ cells and (**f**) CD13^+^ cells detached from CD31^+^ microvessel at the lesion site, 1 week after stroke. (**g**) Representative multiplex RNAscope^™^ F.I.S.H images showing *PDGFβ* and *IGF1* mRNA respective simultaneous expression at the lesion site at 24, 72 h and 1 week after ischemic stroke in VEH and P250 groups. (**h**) Analysis of *PDGFRβ* mRNA spatiotemporal expression at 24, 72 h and 1 week after stroke, shown as ipsilateral/contralateral ratio. (**i**) Analysis of *PDGFRβ/IGF1* mRNA spatiotemporal co-localization at the lesion site 1 week after ischemic stroke in VEH and P250 groups. (**j**) Representative fluorescence images of tdTomato^+^ PDGFRβ^+^ perivascular cells co-immunolabelled with CD13 in the intact brain of PDGFRβ^tdTomato^ transgenic mice 2 weeks after tamoxifen-mediated Cre-recombination. (**k**) Representative fluorescence images of reactive PDGFRα^+^ cells and reactive tdTomato^+^ PDGFRβ^+^ perivascular cells at the lesion site in the brain of PDGFRβ^tdTomato^ transgenic mice, 1 week after ischemic stroke (white arrowheads represent PDGFRα^+^ cells dissociated from tdTomato^+^ cells; yellow arrowheads represent PDGFRα^+^ cell closely interacting with tdTomato^+^ cells). Data are boxplot with min/max, (**b-f**) (*n* = 5–6 animals/group); (**g-i**) (*n* = 3 animals/groups). ^*^P < 0.05/^**^P < 0.01/^***^P < 0.001 compared to P250 group (**(b-f, i)** unpaired two-tailed t-test; (**h**) one-way ANOVA/Tukey’s test). IF, immunofluorescence

Optimal coverage of brain endothelial cells by pericytes is required for vascular integrity [49–51]. PDGFRβ regulates pericyte survival, proliferation, migration, and recruitment to endothelial cells in an angiogenic context [52]. Our previous observations suggest that PDGF-D protective effects were mediated through its action on the vasculature. Therefore, we assessed the impact of increased PDGF-D subacute bioavailability on pericyte-endothelial interactions. This was achieved by co-immunolabeling endothelial cells (CD31) and pericytes (PDGFRβ and CD13). CD13 was used to confirm the identity of pericytes as PDGFRβ is expressed in other perivascular cells [53]. Our analysis demonstrated that PDGF-D increased the coverage of CD31^+^ microvessels by PDGFRβ^+^ pericytes (*P* = 0.0568) (Fig. 4a, b), and reduced the ratio of pericytes that are not attached to endothelial cells (Fig. 4c) at the lesion site, 1 week after stroke. These results were confirmed using CD13 (*P* = 0.0699) (Fig. 4d, e, f). Next, we used RNAscope^®^ multiplex F.I.S.H (Fig. 4g), and found that PDGF-D potently increased the expression of *PDGFRβ* mRNA transcripts (VEH-24 H vs. P250-1 W, *P* = 0.0007; VEH-1 W vs. P250-1 W, *P* = 0.0040) (Fig. 4h), associated with an increased co-expression of *IGF1* mRNA transcripts with *PDGFRβ* mRNA transcripts 1 week after stroke (*P* = 0.0148) (Fig. 4i). This suggests that reactive PDGFRβ^+^ pericytes contribute to IGF1 production at the lesion site after ischemic stroke. IGF1 has been demonstrated to preserve vascular integrity under pathological conditions [54]. Under physiological conditions, IGF1 is expressed in perivascular fibroblasts, which express PDGFRα and PDGFRβ [55, 56]. Therefore, we sought to evaluate the dynamics of pericytes (PDGFRβ^+^ cells) and perivascular fibroblasts (PDGFRα^+^/PDGFRβ^+^ cells) using PDGFRβ^tdTomato^ transgenic mice. We first validated tdTomato abundant and specific expression in perivascular cells, which virtually all expressed CD13, confirming their identity as pericytes as well as recombination efficiency (Fig. 4j). Our analysis revealed that PDGFRα^+^ and perivascular tdTomato^+^ cells (i.e. deriving PDGFRβ^+^ cells) constitute two distinct populations at the lesion site 1 week after ischemic stroke (Fig. 4k). Nonetheless, we observed that reactive PDGFRα^+^ at the lesion site established contacts with the microvasculature by closely wrapping perivascular tdTomato^+^ cells. This indicates that until 1 week after ischemic stroke, PDGFRβ^+^ cells at the lesion site, which is located between the striatum and overlaying cortex, are mainly reactive pericytes and that PDGFRα^+^ cells constitute a distinct population of reactive cells, yet that intimately interacts with reactive PDGFRβ^+^ cells. Our findings demonstrate that PDGF-D subacute induction promotes the interaction of pericytes with the microvasculature after stroke.

### Increasing PDGF-D subacute bioavailability does not alter microvascular immune and barrier properties

**Fig. 5:**
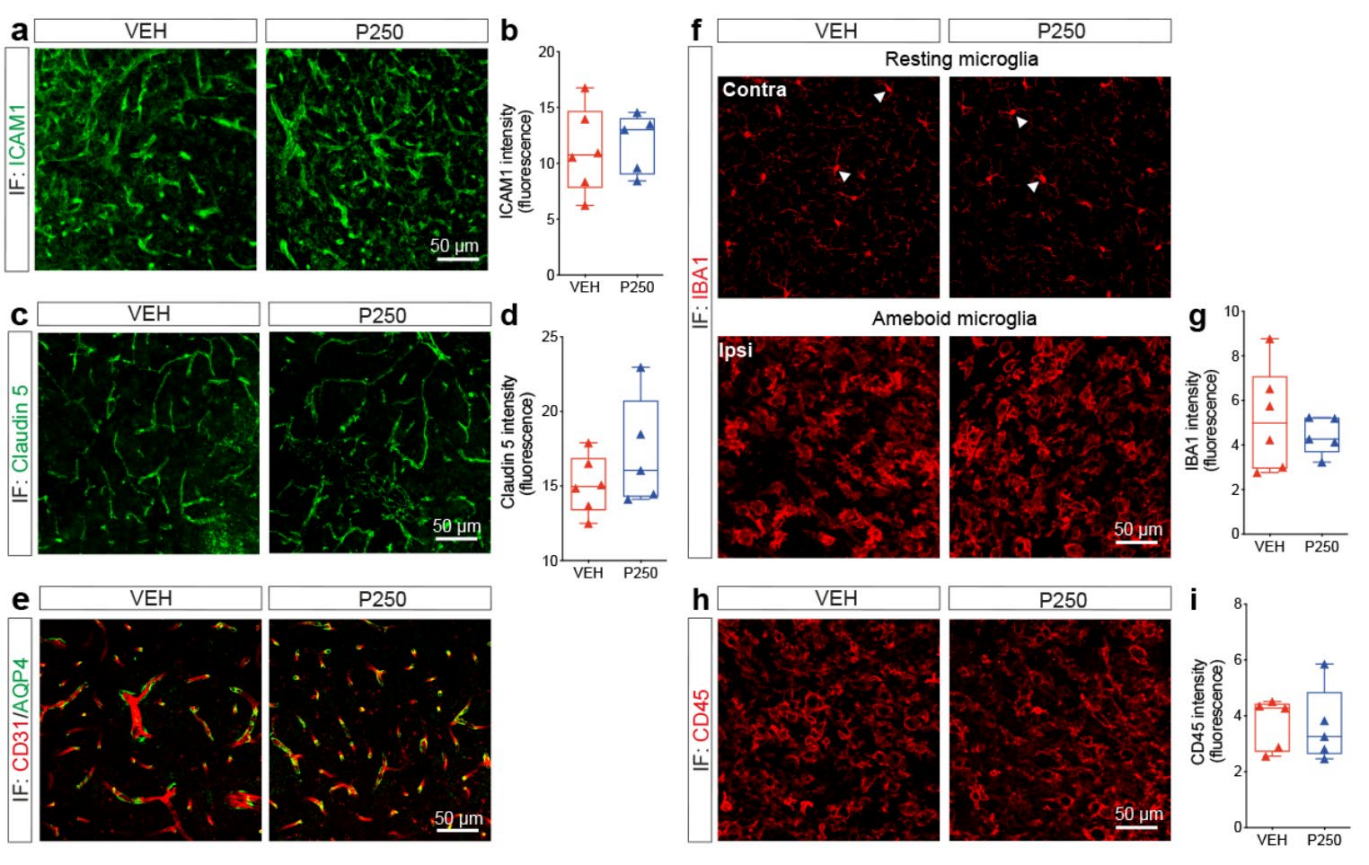
PDGF-D increased subacute bioavailability does not affect microvascular-mediated immune responses. (**a**) Representative fluorescence images of ICAM1 immunolabelling at the lesion site in vehicle (VEH)- or rh-PDGF-D 250 ng/kg (P250)-treated animals, 1 week after ischemic stroke. (**b**) Analysis of ICAM1 expression in VEH and P250 groups, 1 week after ischemic stroke. (**c**) Representative fluorescence images of claudin 5 immunolabelling at the lesion site in VEH and P250 groups, 1 week after ischemic stroke. (**d**) Analysis of claudin 5 expression at the lesion site in VEH and P250 groups, 1 week after ischemic stroke. (**e**) Representative fluorescence images of CD31/AQP4 co-immunolabelling at the lesion site in VEH and P250 groups. (**f**) Representative fluorescence images of IBA1 immunolabelling in the contralateral and ipsilateral hemispheres of VEH and P250 groups, 1 week after ischemic stroke. (**g**) Analysis of IBA1 expression at the lesion site in VEH and P250 groups, 1 week after ischemic stroke (whitehead arrows represent resting ramified microglia in the contralateral hemisphere). (**h**) Representative fluorescence images of CD45 immunolabelling at the lesion site in VEH and P250 groups, 1 week after ischemic stroke. (**i**) Analysis of CD45 expression at the lesion site in VEH and P250 groups, 1 week after ischemic stroke. Data are boxplot with min/max (*n* = 6 animals in VEH and *n* = 5 animals in P250 groups, respectively) ((**b, d, g, i**) unpaired two-tailed test). IF, immunofluorescence; Contra, contralateral; Ipsi, ipsilateral

PDGF-D has been reported to promoting the infiltration of PDGFRβ^+^ macrophages in an experimental model of intracerebral hemorrhage [46]. Therefore, we aimed here to assess the impact of PDGF-D increased subacute availability by analyzing microvascular-mediated immune responses and barrier properties. We assessed first microvascular immune properties by immunolabeling ICAM1 (Fig. 5a). Our analysis indicated that ICAM1 vascular expression at the lesion site remained unchanged 1 week after ischemic stroke upon increasing PDGF-D subacute bioavailability (Fig. 5b). This indicates that PDGF-D did not affect the inflammatory status of the microvasculature at the lesion site. Destabilization of the inter-endothelial cell tight junctions (TJs) contributes to exacerbation of neuroinflammation after ischemic stroke [57]. For this purpose, the status of the TJs was assessed by immunolabeling claudin 5, which is a key junctional protein involved in BBB formation (Fig. 5c). Our analysis demonstrated that claudin 5 expression was preserved in the microvasculature at the lesion site 1 week after ischemic stroke upon increasing PDGF-D subacute bioavailability (Fig. 5d). The barrier properties of brain microvasculature are dependent upon the adequate interaction of endothelial cells with astrocyte-endfeet [58], which was assessed by co-immunolabeling CD31 and AQP4 at the lesion site 1 week after ischemic stroke (Fig. 5e). We found that AQP4 co-localization with CD31^+^ microvessels at the lesion site was preserved 1 week after ischemic stroke upon PDGF-D increased subacute bioavailability. Infiltration of macrophages was next evaluated by immunolabeling IBA1, which marks brain resident microglia as well, thus enabling assessment of overall myeloid cell recruitment (Fig. 5f). Our analysis did not yield any changes in overall IBA1 expression at the lesion site 1 week after ischemic stroke (Fig. 5g). These observations were further confirmed by analyzing CD45 expression (Fig. 5h, i). This indicates that increasing PDGF-D subacute bioavailability does not seem to affect the infiltration and recruitment of immune cells into the lesion site 1 week after ischemic stroke. Our findings suggest that PDGF-D subacute increased bioavailability does not affect the inflammatory and barrier properties of the microvasculature as well as infiltration of immune cells into the brain after ischemic stroke.

### Subacute PDGF-D improves brain repair and neurological recovery after ischemic stroke

**Fig. 6:**
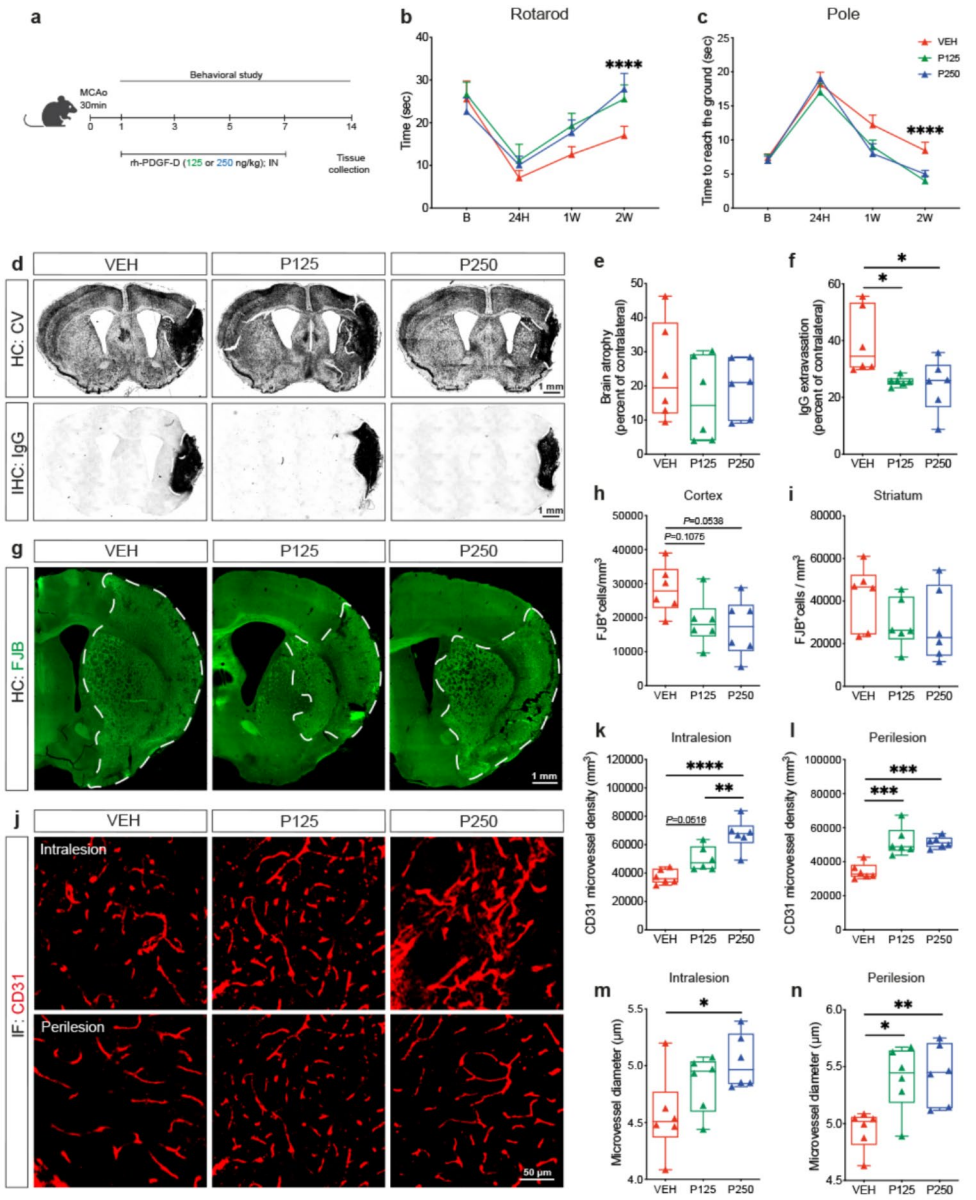
Increased PDGF-D subacute bioavailability mediates sustained recovery after stroke. (**a**) Schematic illustration of the experimental design of rh-PDGF-D intranasal administration 24 h after stroke and repeated every 2^nd^ day until 1 week after stroke, followed by a withdrawal for 1 week before euthanizing mice. (**b**) Analysis of rotarod test performed at baseline, 24 h, 1 and 2 weeks after stroke. (**c**) Analysis of the pole test performed at baseline, 24 h, 1 and 2 weeks after stroke. (**d**) Representative images of cresyl violet histology showing the injury area surrounded by a white dashed line in the ipsilateral hemisphere and IgG immunohistochemistry showing IgG extravasation area in black surrounded by a white dashed line in the ipsilateral hemisphere, 2 weeks after stroke. (**e**) Analysis of brain atrophy represented as percent of contralateral in vehicle (VEH)-, rh-PDGF-D 125 ng/kg (P125)- or rh-PDGF-D 250 ng/kg (P250)-treated animals, 2 weeks after ischemic stroke. (**f**) Analysis of IgG extravasation represented as percent of contralateral in VEH, P125 or P250 groups. (**g**) Representative fluorescence images of FJB^+^ degenerating neurons surrounded by a white dashed line in the ipsilateral cortex and striatum, 2 weeks after ischemic stroke. Stereological analysis of the density of FJB^+^ degenerating neurons in the ipsilateral (**h**) cortex and (**i**) striatum, 2 weeks after ischemic stroke. (**j**) Representative fluorescence images of CD31 immunolabeling in the intralesional and perilesional sites in VEH, P125 or P250 groups, 2 weeks after ischemic stroke. Analysis of the density of CD31^+^ microvessels in the (**k**) intralesional and (**l**) perilesional sites, 2 weeks after ischemic stroke. Analysis of the average diameter of CD31^+^ microvessels in the (**m**) intralesional and (**n**) perilesional sites, 2 weeks after stroke. Data are boxplot with min/max (*n* = 6 animals/group). ^*^P < 0.05/^**^P < 0.01/^***^P < 0.001/^****^P < 0.0001 compared to P125 or P250 groups (**(b, c)** two-way ANOVA/Tukey’s test, **(e-n)** one-way ANOVA/Tukey’s test). IN, intranasal; HC, histochemistry; IHC, immunohistochemistry; IF, immunofluorescence; B, baseline; H, hour; W, week

Our results suggest that subacute PDGF-D induction mediates neurovascular remodeling while preserving functions. Therefore, we aimed next to evaluate whether PDGF-D subacute neuroprotective effects enable long-term structural repair and neurological recovery after ischemic stroke. To this end, active rh-PDGF-D was intranasally infused as specified before, and mice were kept for additional week before being euthanized at 2 weeks after ischemic stroke (Fig. 6a). This strategy was chosen to recapitulate increased PDGF-D subacute endogenous post-stroke regulation. Different neurobehavioral tests were performed to assess motor functions. Using rotarod test to evaluate motor coordination in response to forced motor activity, we found that PDGF-D dose-dependently reduced motor deficits, translated by a higher latency to fall compared to VEH (*P* < 0.0001) (Fig. 6b). Furthermore, using pole test to examine spontaneous locomotor activity, we found that upon increasing PDGF-D subacute bioavailability mice spent less time to reach the ground compared to VEH (*P* < 0.0001) (Fig. 6c). Next, we evaluated whether the neurobehavioral changes were associated with an enhanced post-stroke structural repair. Analysis of cresyl violet histology and IgG immunohistology (Fig. 6d) showed that brain atrophy remained unchanged (VEH:23.92 ± 14.44, P125:15.98 ± 12.29, P250:19.67 ± 8.479) (Fig. 6e), whereas IgG infiltration into the lesion site was reduced (VEH vs. P125, *P* = 0.0219; VEH vs. P250, *P* = 0.0014) (Fig. 6f). Analysis of FJB staining (Fig. 6g) indicated that the increased PDGF-D subacute bioavailability was sufficient to decelerate neuronal degeneration in the cortex (VEH:28420 ± 7058, P125:18834 ± 7168, P250:17166 ± 8539; VEH vs. P125, *P =* 0.1075; VEH vs. P250, *P* = 0.0538) (Fig. 6h), but to a lesser extent in the striatum (VEH:41963 ± 14816, P125:29620 ± 11595, P250:28753 ± 17210) (Fig. 6i), 2 weeks after ischemic stroke. Importantly, analysis of CD31 immunolabeling indicated that microvascular density was maintained until 2 weeks after ischemic stroke upon increasing PDGF-D subacute bioavailability **(**Fig. 6j**)**. This was translated by an enhanced density of CD31^+^ microvessels in the intralesional (VEH vs. P125, *P* = 0.0516; VEH vs. P250, *P* < 0.0001; P125 vs. P250, *P* = 0.0093) (Fig. 6k) and the perilesional (VEH vs. P125, *P* = 0.0003; VEH vs. P250, *P* = 0.0004) (Fig. 6l) sites. Moreover, PDGF-D dose-dependently improved the average diameter of CD31^+^ microvessels in the intralesional (VEH:4.566 ± 0.3619, P125:4.849 ± 0.2492; VEH vs. P250, *P* = 0.0317) (Fig. 6m) and perilesional (VEH vs. P125, *P* = 0.0169; VEH vs. P250, *P* = 0.0097) (Fig. 6n) sites, outlining an improved microvascular structure. These findings suggest that PDGF-D stimulates neurovascular repair and neurological recovery after ischemic stroke.

### PDGF-D does not affect neurogenesis and scar formation associated with ischemic stroke

**Fig. 7:**
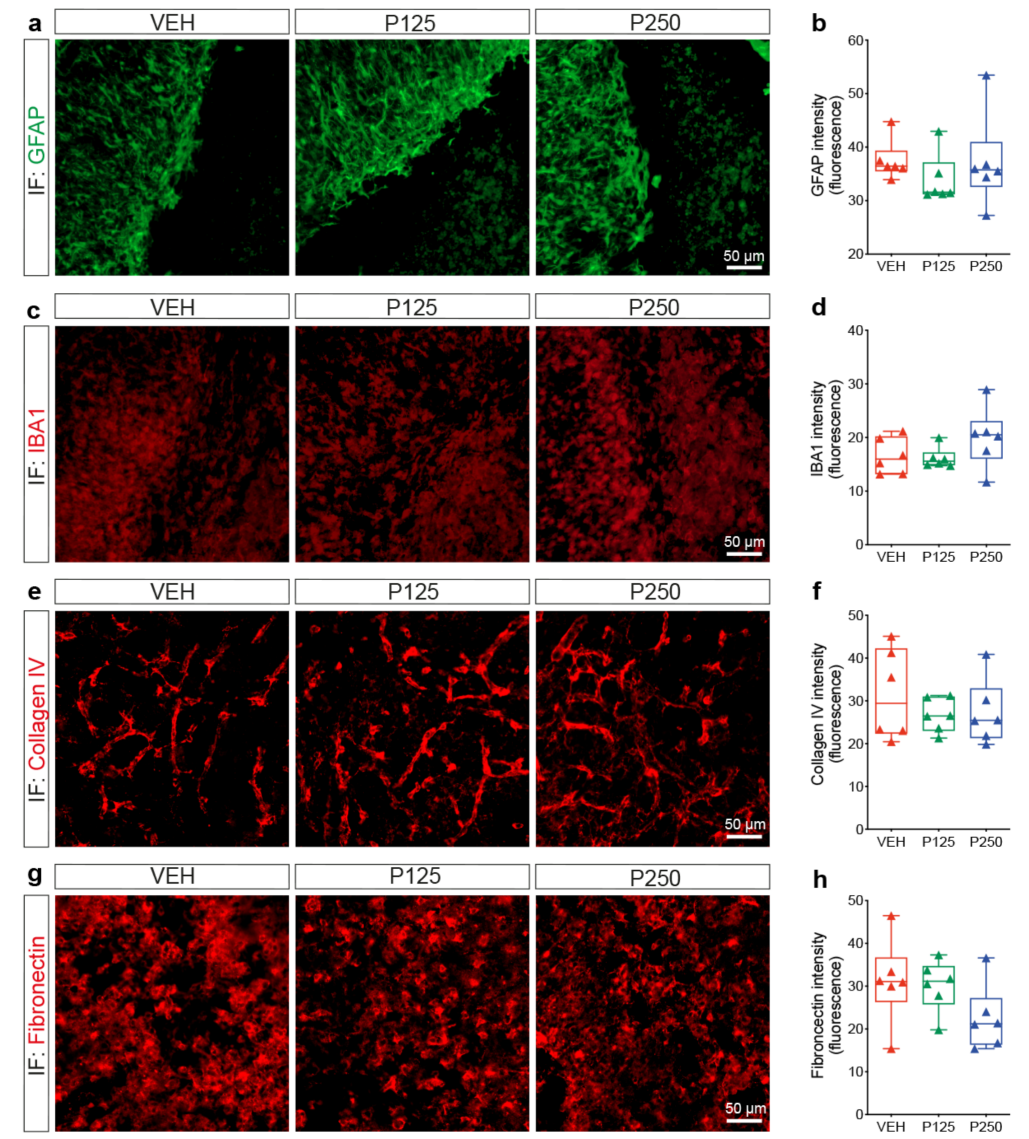
Glial and fibrotic reactions remain unchanged upon PDGF-D subacute brain increase. (**a**) Representative fluorescence images of GFAP immunolabeling at the lesion site in vehicle (VEH)-, rh-PDGF-D 125 ng/kg (P125)- or rh-PDGF-D 250 ng/kg (P250)-treated animals, 2 weeks after ischemic stroke. (**b**) Analysis of GFAP^+^ fluorescence intensity in VEH, P125 or P250 groups, 2 weeks after ischemic stroke. (**c**) Representative fluorescence images of IBA1 immunolabeling at the lesion site in VEH, P125 or P250 groups, 2 weeks after ischemic stroke. (**d**) Analysis of IBA1-immunolabeling fluorescence intensity in VEH, P125 or P250 groups, 2 weeks after ischemic stroke. (**e**) Representative fluorescence images of collagen IV immunolabeling at the lesion site in VEH, P125 or P250 groups, 2 weeks after ischemic stroke. (**f**) Analysis of collagen IV-immunolabeling fluorescence intensity in VEH, P125 or P250 groups, 2 weeks after ischemic stroke. (**g**) Representative fluorescence images of fibronectin immunolabeling at the lesion site in VEH, P125 or P250 groups, 2 weeks after ischemic stroke. (**h**)Analysis of fibronectin^+^ fluorescence intensity in VEH, P125 or P250 groups, 2 weeks after stroke. Data are boxplot with min/max (*n* = 6 animals/group) ((**b, d**) Kruskal-Wallis/Dunn’s test; (**f-h**) one-way ANOVA/Tukey’s test). IF, immunofluorescence

Ischemic stroke is associated with activation of neurogenesis in the neurogenic niches [59, 60]. Neuronal precursor cells (NPCs) originating from the subventricular zone (SVZ), and lateral ventricle (LV) migrate to the injury zone to differentiate into mature neurons as an attempt to replace the lost cells [61–63]. A sub-population of PDGFRβ^+^ stem cells were identified in the SVZ/LV [64, 65]. We aimed to explore whether increasing PDGF-D subacute brain bioavailability affects the neurogenic responses after ischemic stroke. For this purpose, we assessed neurogenesis rate by immunolabeling DCX, a marker of NPCs, 1 week after stroke, and found that PDGF-D did not affect DCX^+^ cell density in the SVZ/LV (VEH:11739 ± 4435, P250:20556 ± 10,095). We evaluated next the capacity of NPCs to give rise to neurons at the lesion site by immunolabeling TUJ1, a marker of immature neurons. Our analysis indicated that increasing PDGF-D subacute bioavailability did not affect the density of TUJ1^+^ cells in the peri-lesion site 1 week (VEH:9098 ± 4352, P250:17290 ± 11923) and 2 weeks (VEH:7125 ± 4389, P250: 24994 ± 26918) after ischemic stroke (data not shown). These findings indicate that subacute PDGF-D induction does not modulate stroke-mediated neurogenic responses. Tissue repair after ischemic stroke is associated with the formation of glial and fibrotic scars in the chronic phase [66]. Therefore, we analyzed the activation pattern of astrocytes and microglia upon increasing PDGF-D subacute bioavailability by immunolabeling GFAP and IBA1, respectively (Fig. 7a, c). Our results showed that PDGF-D did not affect the reactivity of GFAP^+^ cells (Fig. 7b) nor that of IBA1^+^ cells (Fig. 7d) at the lesion site 2 weeks after ischemic stroke. Reactive astrocytes could adopt either an isomorphic phenotype associated with progressive neurodegenerative disorders [67] or anisomorphic phenotype associated with segregation of the intact region upon acute brain injuries [68]. PDGF-D increased subacute bioavailability did not alter the projections of reactive GFAP^+^ astrocytes, indicating a preservation of the anisomorphic phenotype 2 weeks after ischemic stroke (VEH:36.41 ± 1.078; P125:37.833 ± 1.115; P250:37.638 ± 1.661). The ventricle size, which was calculated as a ratio ipsilateral over contralateral, remained unchanged as well (VEH:1.109 ± 0.118; P125:1.073 ± 0.075; P250:1.075 ± 0.052). Afterwards, we investigated the deposition of fibrotic proteins, namely collagen IV and fibronectin [69]. Our analysis showed that collagen IV reactivity at the basal membrane of the vasculature (Fig. 7e) remained unchanged 2 weeks after stroke (VEH:31.46 ± 10.55, P125:26.66 ± 3.919, P250:27.28 ± 7.546) (Fig. 7g) upon increasing PDGF-D subacute bioavailability. Fibronectin reactivity was not apparent 1 week after stroke, and its deposition (Fig. 7h) was not affected by PDGF-D 2 weeks after ischemic stroke (VEH:31.25 ± 9.891, P125:30.16 ± 5.996, P250:22.53 ± 7.591) (Fig. 7i). Our results indicate that increasing PDGF-D subacute availability does not affect the neurogenic responses nor glial and fibrotic reactivities after ischemic stroke.

### PDGF-D promotes pericyte survival, migration and release of vascular protective factors

**Fig. 8:**
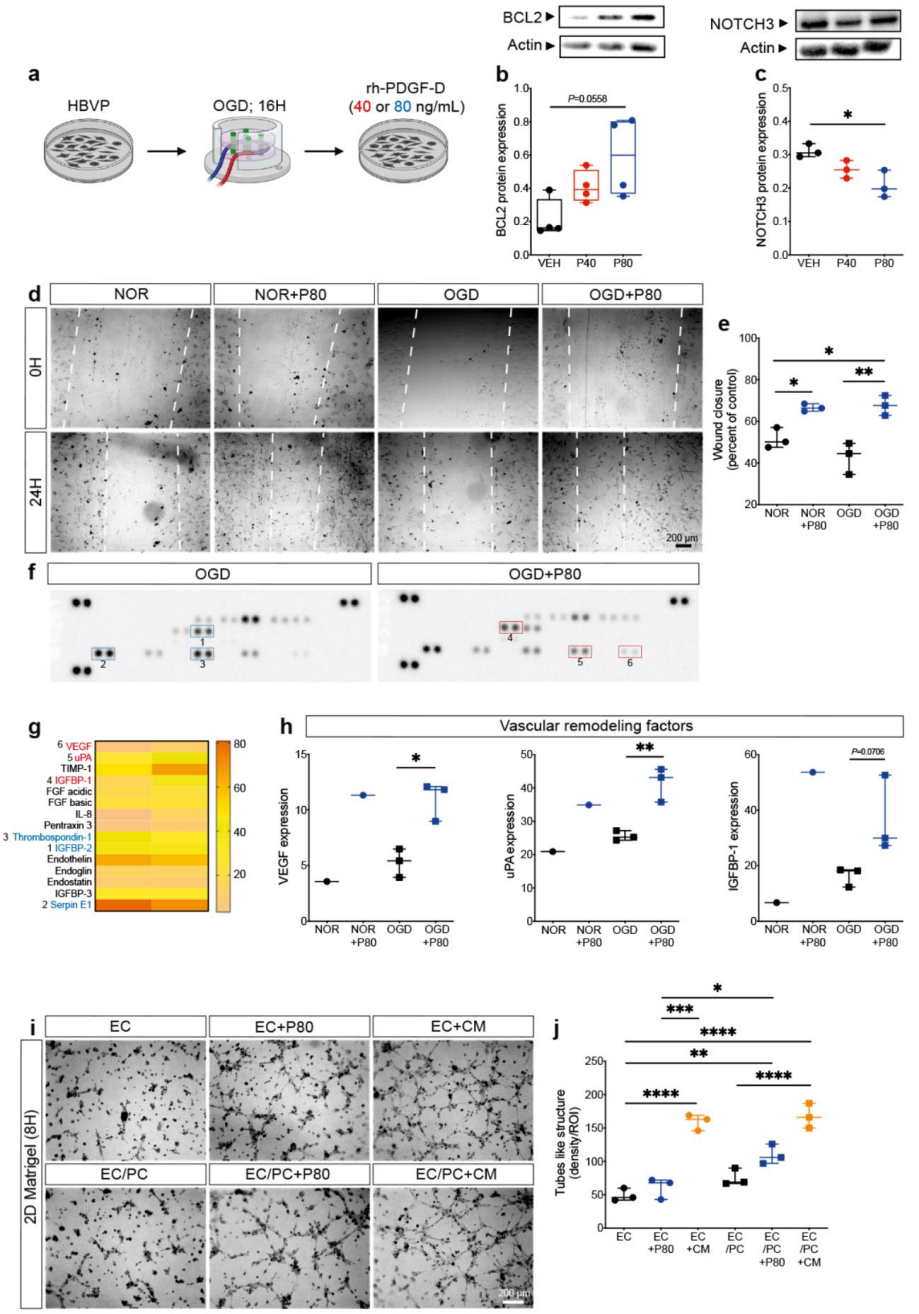
Brain pericyte survival and vascular protective properties are stimulated by PDGF-D. (**a**) Schematic illustration of the experimental design of primary human brain vascular pericytes (HBVP) exposition to oxygen and glucose deprivation (OGD) followed by rh-PDGF-D stimulation. (**b**) Western blot analysis showing BCL2 expression in vehicle (VEH)-, rh-PDGF-D 40 ng/mL (P40)- or rh-PDGF-D 80 ng/mL (P80)-treated HBVP, 24 h after OGD. (**c**) Western blot analysis showing NOTCH3 expression in VEH, P40 or P80 groups, 24 h after OGD. (**d**) Representative brightfield images of VEH and P80 groups, immediately upon or 24 h after wound formation (white dashed lines) under normoxic (NOR) or OGD conditions. (**e**) Analysis of wound closure of VEH and P80 groups under NOR or OGD conditions at 24 h, represented as percent of control (baseline, 0 h). (**f**) Representative images of the proteome Profiler human angiogenesis array membranes analyzing the expression of factors implicated in vascular remodeling in VEH and P80 groups exposed to NOR or OGD, 24 h after stimulation. Blue rectangles highlight downregulated factors and red rectangles highlight upregulated factors. (**g**) Heatmap illustration showing differentially regulated factors in OGD-exposed HBVP upon PDGF-D stimulation. (**h**) Analysis of VEGF, uPA and IGFBP1 expression in NOR- and OGD-exposed HBVP upon stimulation of PDGF-D. (**i**) Representative brightfield images of tubes formed by immortalized human brain endothelial cells (iHBEC) without treatment (EC), treated with P80 (EC + P80) or with CM of OGD-exposed HBVP (PC) stimulated with P80 (EC + CM), or co-cultured with HBVP (PC) without treatment (EC/PC), treated with P80 (EC/PC + P80) or with CM of OGD-exposed PC stimulated with P80 (EC/PC + CM), 8 h after plating cells on ECM. **(j)** Analysis of the density of tubes formed under the different conditions. (**b-e, j**) Data are boxplot with min/max (*n* = 3–4/groups). ^*^P < 0.05/^**^P < 0.01/^***^P < 0.001/^****^P < 0.0001 compared to other different condition (**(b)** Kruskal-Wallis/Dunn’s test, (**c, e, j**) one-way ANOVA/Tukey’s test or (**h**) unpaired two tailed t-test). H, hour

Our hereabove findings indicate that PDGF-D maintains vascular integrity by protecting pericytes upon ischemic stroke. Therefore, we aimed to elucidate the underlying mechanisms using cell-based assays. For this purpose, HBVP were exposed to OGD to mimic ischemia and reperfusion-like conditions in vitro. Normoxia- and OGD-exposed HBVP were stimulated with PDGF-D at 40 ng/mL or 80 ng/mL for 24 h (Fig. 8a). Interestingly, PDGF-D stimulation increased the expression of the anti-apoptotic protein BCL2 (VEH:0.1623 ± 0.1843, P40:0.3931 ± 0.1815, P80:0.5996 ± 0.4317) (Fig. 8b) [70], and reduced at high dose the expression of NOTCH3 (*P* = 0.0147) (Fig. 8c), which mediates pericyte pro-fibrotic properties [71, 72]. Moreover, we evaluated the impact of PDGF-D stimulation on pericyte migration, which is required for proper recruitment to endothelial cells [73], using the wound healing assay (Fig. 8d). Our analysis showed that PDGF-D accelerated the wound closure under normoxic conditions and rescued OGD-mediated impaired wound closure, 24 h after stimulation (NOR vs. NOR + P80, *P* = 0.0322; OGD vs. OGD + P80, *P* = 0.0017; NOR vs. OGD + P80, *P* = 0.0227) (Fig. 8e), outlining a restored pericyte motility. Next, we profiled the release of vascular remodeling factors by HBVP exposed to OGD with and without PDGF-D stimulation using proteome profiler™ human angiogenesis array (Fig. 8f). Our analysis showed that PDGF-D stimulation modulated the expression of several molecules involved in fine-tuning angiogenesis (Fig. 8g). More specifically, PDGF-D stimulation increased the expression of pro-angiogenic factors, namely VEGF (*P* = 0.0102), urokinase-type plasminogen activator (uPA) (*P* = 0.0066) involved in endothelial cell migration [74, 75], and insulin-like growth factor-binding protein (IGFBP)1 (*P* = 0.0706) involved in fine-tuning the angiogenic response [76, 77] (Fig. 8h). In addition, PDGF-D reduced the expression of molecules implicated in stabilizing the angiogenic vasculature, namely thrombospondin (TSP)1 (OGD:40.28 ± 22.68, OGD + P80:27.9 ± 4.669), and serpin E1 (OGD:80.76 ± 3.946, OGD + P80:70.5 ± 12.42). Finally, we investigated the consequences of PDGF-D stimulation on functional angiogenesis using different HBVP and iHBEC culture strategies. Pericyte-endothelial co-culture was associated with an enhanced formation of tube-like structures, which were increased upon stimulation with PDGF-D (EC vs. EC/PC + P80, *P* = 0.0023) or incubation with CM (EC vs. EC + CM, *P* < 0.0001) (Fig. 8i, j). Interestingly, incubation of iHBEC with CM increased the formation of tube-like structures (*P* < 0.0001), whereas iHBEC direct stimulation with PDGF-D had no impact (*P* = 0.9066). This indicates that PDGF-D acted on pericytes and not on endothelial cells. Our results suggest that PDGF-D promotes pericyte survival and association with endothelial cells under ischemia and reperfusion-like conditions while stimulating stable vascular remodeling.

## Discussion

Ischemic stroke triggers an angiogenic response as an attempt to promote injured tissue neovascularization and to subsequently enable structural repair and neurological recovery [11, 13]. A high rate of vascularization and elevated levels of pro-angiogenic factors are associated with mild brain injury after ischemic stroke [11, 13]. These observations highlighted the therapeutic promises of stimulating injured tissue neovascularization after ischemic stroke through angiogenesis [11, 13]. Unfortunately, potent pro-angiogenic factors that act directly on endothelial cells, such as VEGF, are associated with an elevated risk of hemorrhages due to an excessive vascular destabilization [11, 13]. Furthermore, nascent microvasculature are often immature due to a poor coverage with pericytes [13]. Microvascular homeostasis critically depends upon pericyte interaction with endothelial cells, governed by different mechanisms, including PDGFs-PDGFRβ axis [12, 19–21, 78–80]. Herein, we show that PDGF-D expression is induced in the subacute phase of ischemic stroke in brain endothelial cells. It is noteworthy to indicate that concomitantly with PDGF-D endothelial induction, perivascular PDGFRβ was transcriptionally induced at the lesion site. To evaluate the biological relevance of PDGF-D subacute endogenous induction, its expression was downregulated using siRNA delivered non-invasive into the brain via the intranasal route. Attenuation of PDGF-D expression exacerbates neuronal loss as well as reduced vascular integrity and density at the lesion site 1 week after ischemic stroke. Notably, we observe an increased rate of microvascular stalls that have been previously shown to impede vascular patency and CBF restoration after ischemic stroke, partly due to pericyte dysfunction, thus accelerating secondary injury progression and worsening overall outcomes [81, 82]. Our findings unveil a previously undescribed role of PDGF-D in regulating angiogenesis after ischemic stroke, while mediating preservation of microvascular structure and function, consequently improving structural and functional outcomes.

Expression of various PDGF ligands potently increases after ischemic stroke [48, 83]. PDGF-AB/BB elevated levels are independently associated with a lower risk of recurrent vascular events in stroke patients [83]. Endothelial PDGF-B acts on PDGFRβ^+^ pericytes to promote stability of the angiogenic vasculature [22, 84]. Deregulation of PDGFRβ signaling aggravates brain damage and neurological deficits after ischemic stroke [19, 21, 23, 24]. However, activation of PDGFRα^+^ perivascular astrocytes exacerbates microvascular impairments after ischemic stroke [26, 85]. A subpopulation of astrocytes express basal levels PDGFRβ, in comparison to perivascular cells, but it remains unknow whether PDGFRβ is expressed in this subpopulation as a homodimer or form a heterodimer with PDGFRα [25]. PDGF-BB homodimer or PDGF-AB heterodimer could activate PDGFRα [27, 84], but PDGF-D forms exclusively a homodimer ligand that has a high specific affinity for PDGFRβ homodimers expressed in perivascular cells [25]. Under pathological conditions, PDGF-D stimulates vascular maturation in peripheral organs via regulation of the functions of perivascular cells [30, 86]. Herein, we report that increasing PDGF-D subacute bioavailability attenuates neuronal loss, reduces microvascular permeability, and improves neurological recovery 1 week after ischemic stroke. Attenuation of neuronal loss was not associated with an improved post-stroke neurogenic response [87], suggesting that PDGF-D subacute induction promotes tissue protection rather than stimulating regeneration. Importantly, we demonstrate that PDGF-D-mediated protective effects in the subacute phase are maintained over time and enable a sustained long-term recovery, assessed herein 2 weeks after ischemic stroke. Some reports have indicated that PDGF-D might regulate inflammation by acting on PDGFRβ^+^ macrophages and PDGFRβ^+^ fibroblasts [46, 88]. Herein, we show that increasing PDGF-D subacute abundance does not affect astrocytic and microglial respective reactivity [89]. In a model of chronic cerebral ischemia, PDGF-B/VEGF chronic co-delivery into the brain protects from ischemia by improving brain perfusion, angiogenesis, and pericyte association with the microvasculature [90]. These findings align with our herein reported observations in an experimental mouse model of focal ischemic stroke. However, PDGF-B/VEGF chronic co-delivery induces a potent activation of reactive astrocytes, mediated presumably through PDGF-B action on PDGFRα^+^ perivascular astrocytes. Absence of changes in astrocytic reactivity could be associated with PDGF-D higher affinity for cells abundantly expressing PDGFRβ homodimers, comprising essentially brain pericytes. PDGFRβ^+^ perivascular cells contribute to fibrotic scar formation by secreting fibrotic proteins at the lesion site [15, 21, 91]. PDGFRβ^+^ fibroblasts co-expressing PDGFRα, which are essentially associated with the large pial penetrating vasculature, and PDGFRβ^+^ pericytes, which are essentially associated with parenchymal microvasculature, both have been reported to contribute to fibrotic scar formation after ischemic stroke [56, 92]. Expression of major ECM proteins, such as collagen IV and fibronectin, remains unchanged 1 week after ischemic stroke upon increasing PDGF-D subacute bioavailability. It is noteworthy to mention that the process of glial and fibrotic scar is still incomplete at this stage [15]. However, PDGF-D impact on PDGFRβ^+^ cells other than pericytes could not be excluded, but the changes could not be detected 2 weeks after ischemic stroke. Our findings suggest that PDGF-D induced subacute bioavailability does not affect glial and fibrotic responses.

Brain pericytes play a critical role in fine-tuning the angiogenic response [64]. Deregulation of PDGFRβ signaling in ischemic stroke attenuates pericytes interaction with endothelial cells, leading to injury exacerbation associated with impaired angiogenesis and altered CBF restoration [19]. Transplantation of pericyte-like cells attenuates BBB breakdown and promotes neurological recovery after stroke [20]. Depletion of the regulator of G-protein signaling (RGS)5 increases the ratio of vascular-associated PDGFRβ^+^ pericytes and reduces that of parenchymal reactive PDGFRβ^+^ pericytes after ischemic stroke [93, 94]. The vascular redistribution of PDGFRβ^+^ pericytes upon RGS5 depletion after ischemic stroke enhances angiogenesis, while reducing microvascular permeability. These findings demonstrate that rescuing pericyte association with the microvasculature promotes stable angiogenesis after ischemic stroke. Our results indicate that PDGF-D improves the density of the microvasculature, which exhibits reduced stalls at the lesion site, 1 week after ischemic stroke. Notably, PDGF-D-mediated vascular structural integrity are maintained for 2 weeks after ischemic stroke, outlining the formation of a structurally stable new mircovascular network. Interestingly, subacute PDGF-D induction improves overall brain perfusion, indicating the observed structural responses are accompanied by an improved functionality. PDGF-D enhanced subacute bioavailability improves endothelial cell-pericyte interaction, which is essential for vascular stabilization and BBB maintenance [3]. Indeed, PDGF-D increases the ratio of the microvasculature covered by PDGFRβ^+^ and CD13^+^ pericytes at the lesion site, while reducing the ratio of reactive detached PDGFRβ^+^ and CD13^+^ cells. Expression of IGF1, which has been reported to provide protection after stroke by improving CBF and BBB function, is induced in reactive PDGFRβ^+^ pericytes upon increasing PDGF-D brain abundance [54, 95]. Furthermore, IGF1 regulates the angiogenic response by fine-tuning VEGF signaling to enable stabilization of the newly formed microvasculature [96]. Notably, expression of the key junctional protein claudin 5 and astrocyte-endfeet association with the microvasculature remain both unaltered, indicating that BBB properties are preserved despite the active angiogenic response mediated by PDGF-D [57]. Our findings suggest that PDGF-D rescues pericyte-endothelial cell interaction that contributes to the maintenance of the structural and functional integrity of the microvasculature after ischemic stroke.

As mentioned, PDGFRβ plays a central role in regulating pericyte functions. Using cell-based assays, we show that PDGF-D stimulation improves the survival of human brain pericytes exposed to ischemia and reperfusion-like conditions by increasing BCL2 expression. Furthermore, PDGF-D reduces the expression of NOTCH3, which is involved in regulating the quiescence of pericytes when optimal endothelial cell coverage is achieved [97, 98]. Aggregation of NOTCH3 extracellular domain (ECD) in pericytes is implicated in the etiology of cerebral autosomal dominant arteriopathy with subcortical infarcts and leukoencephalopathy (CADASIL) [99]. Indeed, an excessive NOTCH3 activity in pericytes impairs CBF in CADASIL. Furthermore, NOTCH3 promotes fibrosis and inflammation by stimulating the transition of reactive pericytes towards a pro-fibrotic phenotype in various organs [71, 72]. These reports suggest that attenuation of NOTCH3 expression upon PDGF-D stimulation improves the survival of pericytes exposed to ischemia and reperfusion-like conditions, and alleviates their pathological pro-fibrotic transition. Moreover, we show here that PDGF-D stimulation enhances the migratory capabilities of pericytes exposed to ischemia and reperfusion-like conditions. PDGF-D stimulation enriches the secretome of pericytes exposed to ischemia and reperfusion-like conditions by molecules involved in mediating vascular remodeling, namely IGFBP1, uPA, and VEGF, while reducing the expression of molecules involved in inhibiting angiogenesis, namely TSP1, IGFBP2, and serpin E1. IGFBPs regulates the biological action of IGFs [100], and IGFBP1 promotes angiogenesis by increasing nitric oxide (NO) production while improving vascular repair [74, 101, 102]. Our observations are in line with previous reports showing that IGF1/IGFBP1 intranasal delivery is protective after ischemic stroke [103]. uPA promotes angiogenesis by regulating VEGF receptor (VEGFR)1/2 expression in endothelial cells [76]. TSP1 inhibits angiogenesis by negatively regulating NO pathway [102, 104]. TSP1 expression increases after ischemic stroke and is thought to aim at avoiding an aberrant angiogenesis [105]. Serpin E1 inhibits VEGF-mediated VEGFR2 activation to maintain a controlled angiogenic response [106]. These results suggest that PDGF-D-stimulated brain pericytes release pro-angiogenic as well as vascular stabilizing factors to fine-tune the angiogenesis response, leading to the formation of stable new microvascular network. Indeed, stimulation of pericyte-endothelial cell co-culture with PDGF-D, or endothelial cells monoculture with the secretome of PDGF-D-stimulated brain pericytes, increases the density of tube formation, indicative of angiogenesis activation. Brain endothelial cells do not respond to PDGF-D, indicating that the latter effects are associated with its direct action on pericytes.

## Conclusion

Herein, we provide additional evidence outlining the potential of pericytes as potential targets to develop efficient and safe pro-angiogenic therapies for ischemic stroke. Our results demonstrate that PDGF-D expression is induced in brain endothelial cells in the subacute phase of ischemic stroke along with PDGFRβ, and its expression contributes to the spontaneous protection of neurovascular functions by rescuing the vascular protective properties of pericytes. Our results suggest that PDGF-D subacute non-invasive brain delivery represents a promising therapeutic avenue to stimulate structural repair and neurological recovery after ischemic stroke.

## Data Availability

Materials are available from the corresponding author on reasonable request.
